# Characterisation of nanomaterial hydrophobicity using engineered surfaces

**DOI:** 10.1007/s11051-017-3804-z

**Published:** 2017-03-20

**Authors:** Cloé Desmet, Andrea Valsesia, Arianna Oddo, Giacomo Ceccone, Valentina Spampinato, François Rossi, Pascal Colpo

**Affiliations:** 1 Directorate Health, Consumer and Reference Materials, Consumer Products Safety Unit, Via E. Fermi, 2749. TP 125, 21027 Ispra, VA Italy; 2 IMEC, MCACSA, Kapeldreef 75, 3001 Leuven, Belgium

**Keywords:** Nanomedicine, Nanomaterials, Hydrophobicity, Measurement method

## Abstract

Characterisation of engineered nanomaterials (NMs) is of outmost importance for the assessment of the potential risks arising from their extensive use. NMs display indeed a large variety of physico-chemical properties that drastically affect their interaction with biological systems. Among them, hydrophobicity is an important property that is nevertheless only slightly covered by the current physico-chemical characterisation techniques. In this work, we developed a method for the direct characterisation of NM hydrophobicity. The determination of the nanomaterial hydrophobic character is carried out by the direct measurement of the affinity of the NMs for different collectors. Each collector is an engineered surface designed in order to present specific surface charge and hydrophobicity degrees. Being thus characterised by a combination of surface energy components, the collectors enable the NM immobilisation with surface coverage in relation to their hydrophobicity. The experimental results are explained by using the extended DLVO theory, which takes into account the hydrophobic forces acting between NMs and collectors.

**Electronic supplementary material:**

The online version of this article (doi:10.1007/s11051-017-3804-z) contains supplementary material, which is available to authorized users.

**Graphical abstract:**
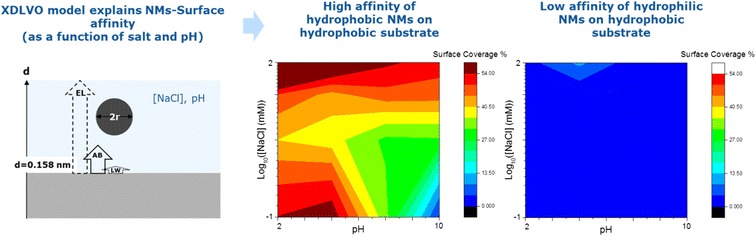
Determination of hydrophobicity character of nanomaterials by measuring their affinity to engineered surfaces.

## Introduction

Engineered nanomaterials (NMs) are widely used in a large number of consumer and industrial products (Vance et al. 2015) and in many healthcare-related products such as biosensors, Point of Care devices and nanomedicines (Gessner et al. 2000). The understanding of their interactions with biological systems is of great importance for the assessment of risk and benefit associated with their use. For this purpose, the physico-chemical properties of NMs are essential parameters to be determined since they dictate their behaviour in a given environment (water, buffer, biological fluid etc.) through different surface molecular interactions. For instance, the physico-chemical properties influence the formation of the so-called protein corona around the nanoparticles upon contact with serum proteins in the blood stream. This corona plays an important role in the interactions of nanomedicines with cells and organs, determining their pharmacokinetic and pharmacodynamics profiles (Gessner et al. 2000; Monopoli et al. 2012; Saptarshi et al. 2013; Tenzer et al. 2013; Walczyk et al. 2010).

Surface hydrophobicity plays a critical role in various biological processes, including protein adsorption and denaturation (Gessner et al. 2000), interaction with biological membranes (Shima et al. 2013) or cellular uptake, and it is potentially related to an increase in toxicity (Chompoosor et al. 2010; Zhu et al. 2012), immune response (Moyano et al. 2012) or haemolytic effect (Saha et al. 2014). It has been demonstrated that the NM hydrophobicity has a direct influence on the stability and bio-distribution of nanovectors (Gessner et al. 2000; Jones et al. 2014) and thus is a key property to be controlled, especially for nanomedicine application. Different methods are currently available for NM hydrophobicity characterisation (Xiao and Wiesner 2012), e.g. surface adsorption assays (Doktorovova et al. 2012), NM relative affinity for reference phases and hydrophobic interaction chromatography (Carstensen et al. 1991). Nevertheless, these methods involve expensive and time-consuming analytical techniques. The development of fast methods for NMs hydrophobicity characterisation would then be of a great interest for NMs and nanomedicine producers providing a meaningful reduction of cost per analysis.

A comprehensive theory explaining the intermolecular forces between two surfaces interacting in a given liquid is the extended Derjaguin, Landau, Verwey, Overbeek theory (van Oss 1993) (XDLVO), where the total interaction energy *G* ^tot^ is the sum of electrostatic, acid-base (AB) and Lifshitz-Van der Waals (LW) interaction contributions. According to the XDLVO theory, the three potentials depend, among on other parameters, on the relative distance between the two interacting surfaces. By knowing the surface energy components and the electrostatic property (i.e. the surface charge) for the two surfaces, the interacting potential can be calculated enabling to predict if the two surfaces will experience attractive or repulsive forces in the given medium.

The AB and LW surface energy components for a material contain the information on the electronic properties of the surface: electrons can be exchanged between two surfaces (as in the electron donor-acceptor mechanism) or generate dipoles, if surrounded by a polar medium. They both contain the information on the hydrophobicity of a surface, which affects the affinity of the given surface for water medium. Hydrophobic surfaces have in general extremely low surface energy components (both the LW and the AB components) since their electrons are strongly bound (as in case of the fluorinated materials) and they naturally tend to attract each other in a polar medium. This is the reason why a hydrophobic colloid is very unstable in water when “hydrophobic” attractive forces overcome the repulsive electrostatic forces.

In this study, we describe a new method for the characterisation of nanoparticle hydrophobicity. The detection platform is based on the measurement of the affinity between NMs and a fluorinated hydrophobic surface, modified by a layer-by-layer (LBL) deposited polyelectrolyte (PE) in order to tune the surface properties and to allow long-range hydrophobic interaction to occur. As a negative control, a hydrophilic surface modified by PE layers is used. We show that these platforms allow discriminating between hydrophilic and hydrophobic NMs, the latter showing a higher affinity for the PE-modified hydrophobic surface. The experimental results are then explained by the XLDVO theory, which enables to predict the affinity of the hydrophobic NMs for the hydrophobic platform in different experimental conditions.

## Materials and methods

### Nanoparticle characterisation

Polystyrene particles (Polybead® Microspheres 0.20 μm, Polysciences) were used as model hydrophobic NMs. The same types of polystyrene particles modified with carboxyl groups (Polybead® Carboxylate Microspheres 0.20 μm, Polysciences) were used as a hydrophilic model.

Particle size distribution was measured by dynamic light scattering (DLS) using a Zetasizer Nano-ZS instrument (Malvern Instruments Ltd., UK) with temperature control (24.9 °C). Measurements of each sample were performed in duplicate with an equilibration step of 120 s. Acquisition time was 80 s. The software was set to automatic acquisition mode. Hydrodynamic diameters were calculated using the internal software analysis. Zeta potential was measured using the same instrument and recorded in a DTS1060C disposable cell with an equilibration time of 120 s. Measurements were done just after pH measurement. A Smulochowski model with a F(Ka) of 1.5 was used.

To confirm the difference in hydrophobicity of the different types of NM, their contact angles with water were measured. Briefly, the original dispersion provided by the supplier was centrifuged and washed in a solution of ethanol/water (30:70) for three times in order to remove the surfactants which might interact with wettability measurements. The NMs were then dispersed in ultrapure water and left to dry on the substrate to create a homogenous layer made of colloids. The contact angle measurements were performed with Milli-Q water as probe liquid, at room temperature. Also, in order to measure the NM dispersive (LW) component of the surface free energy, the contact angles with α-bromonaphtalene were measured with the same method.

Octanol/water partition was also done in order to evaluate the nanomaterials’ hydrophobicity/hydrophilicity. Briefly, 2 ml of octanol and 2 ml of water were added to a glass bottle; the octanol volume stays on top of the water being lighter and totally immiscible. A solution of 100 μl PS particles on the one hand and PS-COOH particles on the other hand were then added to the bi-phase solution at a concentration of 0.4% solid content. The bottle was strongly shaken in order to favour the dispersion of the NMs in the two media. The presence of NMs in the two phases was then observed by naked eyes.

### Surface preparation

Silicon wafers (Si(100), diameter, 50 mm; resistivity, 1–20 Ω cm) supplied by ITME (Warsaw, Poland) were used as the substrate for the whole study. Before modification, the wafers were washed with ethanol and water and dried under nitrogen flow.

#### Plasma polymer deposition

The silicon substrate was modified by different layer depositions in order to tune the surface hydrophobicity. A polytetrafluoroethylene coating was plasma deposited to generate a hydrophobic surface. The deposition was performed using pure octofluorocyclobutane (C_4_F_8_) as the gas precursor at a pressure of 3.5 Pa (27 mTorr), applying a power of 142 W for 5 min (Ruiz et al. 2007). Plasma-polymerised acrylic acid was deposited as a hydrophilic surface, using acrylic acid as precursor at a pressure of 2.1 Pa (16 mTorr), applying a bias power of 400 W for 5 min (Ruiz et al. 2007).

#### Polyelectrolyte layer-by-layer deposition

In order to tune the surface hydrophobicity, a LBL deposition of two polyelectrolytes was performed. The plasma-modified substrates were incubated for 2 min in poly(diallyldimethylammonium chloride) (PDDA) 2% solution in water or in poly(sodium 4-styrene sulfonate) (PSS) 2% in water for the self-assembly deposition of each polyelectrolyte LBL, starting from PDDA (positively charged) and alternating with PSS (negatively charged). After each step, the substrate was rinsed with Milli-Q water and dried under nitrogen flow.

### Surface characterisation

In order to have a complete characterisation of the surfaces, different techniques have been used.

Thickness and refractive index of each deposited layer were measured by Ellipsometry (Vase VUV™ J.A. Woollam Co.). All measurements were performed in air at room temperature for different angles of incidence (between 40° and 70°) with a step width of 0.5° and a low-capacity laser with the wavelength *λ* = 554.3 nm used as a light source. Conventional polarizer-compensator-sample-analyser (PCSA) null-ellipsometric procedure was used to obtain maps of the Δ and ψ angles. The thickness and the complex refractive index were calculated from these two angle maps by point-by-point modelling using the software provided with the ellipsometer, using a two-layer model with the silicon wafer as first layer and a Cauchy layer as the second.

XPS measurements were carried out with an ultra-axis spectrometer (Kratos Analytical Ldt., Manchester, UK) equipped with a monochromatic Al Kα source (hν = 1486.6 eV), operated at 150 W with a spot of 100 μm in diameter. The base pressure was better than 3 × 10^−9^ mbar, and the analysis pressure better than 10^−8^ mbar. Survey spectra (0 to 1150 eV binding energy (BE) range) were collected at a 90° take-off angle (with respect to the sample surface) and with a pass energy of 160 eV. High-resolution spectra were recorded at the same conditions but with a pass energy of 20 eV. Surface charge was compensated by a magnetic charge compensation system, and the energy scale was calibrated by setting the C1s hydrocarbon peak to 285 eV. For each sample, at least three measurements were carried out in a non-superimposing region to investigate the film uniformity.

Data were processed using the Vision 2 software (Kratos Analytical). Curve fitting of C1s peaks were performed using the same initial conditions and inter-peak constraints for each spectra. The Gaussian to Lorentzian mix was varied between 0.7 and 0.9, while the full-width half maximum (FWHM) was kept constant. The area of the β-shifted carbon was constrained to be equal to that of the COOH/R component. The position of the C–O and C=O components were fixed at 1.35–1.5 eV and 2.7–2.85 eV from the CH or C–C component, respectively.

ToF-SIMS analysis was conducted using a reflection-type TOF-SIMS IV spectrometer (ION-TOF GmbH, Münster, Germany) equipped with a 25-keV liquid metal ion gun (LMIG) operating with bismuth primary ions. Spectra were acquired in static mode (primary ion fluence <10^12^ ions cm^−2^) in order to preserve the molecular information. During analysis, charging of the surface was compensated using low-energy (~20 eV) electron flood gun. For each samples, four positive and four negative spectra were acquired in the non-superimposing regions. Mass calibration of ToF-SIMS spectra was done by using the hydrocarbon peaks CH^+^ (13 u), CH_3_^+^ (15 u), C_2_H_3_^+^ (27 u), C_3_H_5_^+^ (41 u), C_5_H_7_^+^ (67 u) and C_7_H_7_^+^ (91 u) for positive ion spectra in order to ensure a good relative mass accuracy. Analyses were obtained from square areas of 200 × 200 μm^2^ (1128 × 128 pixels) in high mass resolution burst mode (resolution M/ΔM>6000). Spectral interpretation was carried out using Surface Lab software v6.4 (ION-TOF GmbH, Münster, Germany).

The wettability of the modified substrates was measured using the sessile drop method with a static contact angle Goniometer (GBX Digidrop, France) employing Milli-Q water and α-bromonaphtalene separately as probe liquid at room temperature. In brief, a 2-μl drop of the probe liquid was dropped from a calibrated micro-syringe over each substrate (taken in triplicate) at three different locations, then the nine measurements were then averaged. The contact angle was measured after each step of the surface modification procedure, on the unmodified silicon wafer first then on PTFE and PAA, as well as after each polyelectrolyte layer on the different substrates.

Surface morphology was measured by scanning probe analysis with a commercial atomic force microscope (SMENA head, Solver electronics, NT-MDT, Russia). The positioning system was equipped with a 3-D closed loop, in order to correct the non-linear behaviour of the piezoelectric crystal. The topography measurements were carried out using a standard tapping mode silicon cantilever with a nominal force constant of 5 N/m.

For the collection of force-distance curves, a standard silicon tip mounted on a soft cantilever (force constant 0.01 N/m), with a nominal radius of curvature of 10 nm was used. Briefly, the tip was brought to contact with the surface in tapping mode, by setting the z-piezoelectric at the middle of its maximum extension (the maximum extension was around 6 μm). Then the system was switched to contact mode and the cantilever was moved away from the surface of about 1 μm, then approached to the surface at a constant speed of 1 μm/s and pushed against the surface for about 0.2 μm. The cantilever was then brought back to the original position (1 um above the surface). The cantilever deflection was recorded as a function of the position of the z-piezoelectric for the approach and the retract curve. The cantilever deflection is a direct measurement of the interaction forces occurring between the tip and the surface. In particular, the adhesion force (when present) is measured when the tip is retracted from the surface right after the indentation, making the cantilever deflect downwards (i.e. with a negative deflection value).

Finally, zeta-potential measurements were performed for a range of pH values from 3 to 10 in order to determine the surface charge using an ElectroKinetic Analyser (Anton Paar, Austria) with a rectangular clamping cell suitable for small flat substrates, based on the streaming potential method. Inside the cell, the sample was pressed against a PMMA spacer with seven rectangular channels. Therefore, the measured zeta potential includes a contribution from the PMMA spacer, which can be eliminated by measuring a reference PMMA surface. For this purpose, a PMMA reference curve was also determined by measuring its zeta potential under the same measuring conditions as the one used with the PAA- and PTFE-modified samples. The pH was adjusted by adding 0.1 M HCl or 0.1 M NaOH. The raw zeta-potential values for both samples were measured in a solution of 1 mM KCl and in steps of approximately 0.5 pH units by automatic titration with 0.1 M HCl. To ensure good statistics, four single measurements with alternating flow direction were taken for each stabilised pH. The zeta potential was calculated based on the Helmholtz–Smoluchowsky equation: ζ = (d*U*/d*p*) × (*η/εε*0) × *K*, where ζ is the zeta potential, d*U* is the streaming potential, d*p* is the pressure differential across the sample, *η* is the viscosity of the electrolyte solution, *ε* is the relative dielectric constant of the fluid, *ε*0 is the vacuum permittivity and *K* is the specific electrical conductivity of the electrolyte solution. The corrected zeta-potential (ζ_c_) values for the different samples were obtained by using the equation ζ_c_ = 2 × ζ_sample_ − ζ_PMMA_ for each concerned pH.

### Nanoparticle-binding study

The two model particles were incubated with collector surfaces with tuned properties to determine their binding, resulting from interaction forces between particles and surfaces. In order to tune the electrostatic forces, the experiments were performed under 16 different conditions of salt concentration ([NaCl] = 0/1/10/100 mM) and pH (2/4/7/10) in aqueous solution. The incubation with NMs was done by full immersion of the substrate in the different NM dispersions for 30 min. The surface is then rinsed thoroughly with Milli-Q water and dried under nitrogen flow before being imaged by scanning electron microscopy (SEM).

SEM measurements were performed by a FEI NOVA 600, Dual Beam, using 5 keV acceleration voltage and acquiring secondary electrons. The average size of particles was calculated through ImageJ software, from at least 100 particles. The surface coverage was calculated from the SEM images using the same software.

### Calculation of the acting potential between nanoparticles and collectors

According to the XDLVO theory (van Oss 1993), the total interaction energy *G* ^tot^ between a flat surface and nanoparticles can be expressed as:

1 $$ {G}^{\mathrm{tot}}={G}^{\mathrm{el}}+{G}^{\mathrm{AB}}+{G}^{\mathrm{LW}} $$

where *G* ^el^ *G* ^AB^ and *G* ^LW^ are related to the electrostatic, acid-base and Lifshitz-Van der Waals interactions, respectively. The three potential depends on the distance between the NM and the surface.

Electrostatic interaction energy:

2 $$ {G}^{\mathrm{el}}=\pi \varepsilon {R}_N\left({\zeta}_N^2+{\zeta}_S^2\right)\left(\frac{2{\zeta}_N{\zeta}_S}{\zeta_N^2+{\zeta}_S^2}\times \ln \frac{1+ \exp \left(-\kappa d\right)}{1- \exp \left(-\kappa d\right)}+ \ln \left\{1- \exp \left(-2\kappa d\right)\right\}\right) $$

where *d* is the separation distance between the NM and the surface and *ζ*_*N*_ and *ζ*_*S*_ are the zeta potential of the nanoparticle and the collector surface, respectively. *1/κ* is the double-layer thickness, which is expressed from the equation:

3 $$ \kappa ={\left(\frac{e^2}{\varepsilon kT}\sum { i z}_i{n}_i\right)}^{1/2} $$

where *ε* is the permittivity of the medium, *e* is the charge of electron, *k* is the Boltzmann constant, *T* is the temperature, *z*_*i*_ is the valency of the ions *i*, and *n*_*i*_ is their number per unit volume.

The Lifshitz-Van der Waals *ΔG* ^*LW*^ components to the free energy of interaction between a nanoparticle and surface are calculated following the XDLVO theory:

4 $$ {G}^{\mathrm{LW}}=-\frac{H}{6}\left(\frac{2 r\left( d+ r\right)}{d\left( d+2 r\right)}- \ln \frac{d+2 r}{d}\right) $$

where *d* is the separation distance between NM and surface, and *r* is the nanoparticle’s radius.

*H* is the effective Hamaker constant for the NM-collector-water system, which can be expressed as:

5 $$ H=24\pi {d}^2\left(\sqrt{\gamma_N^{\mathrm{LW}}}-\sqrt{\gamma_w^{\mathrm{LW}}}\right)\left(\sqrt{\gamma_s^{\mathrm{LW}}}-\sqrt{\gamma_w^{\mathrm{LW}}}\right) $$

While the analytical expressions for the electrostatic potential and the Lifshitz-Van der Waals potentials are well known and commonly accepted, the acid-base interaction potential has mainly an empirical formulation based on experimental observations (Boks et al. 2008; van Oss 1993; Wood and Rehmann 2014) and on direct measurements of the interaction potential between two surfaces (sphere-sphere, sphere-plane, plane-plane) in a polar medium or in an electrolytic solution. The *G* ^AB^ includes all those forces, which involve the structural reorganisation of the water molecules around two surfaces, depending on the degree of wettability of the surfaces involved. For a sphere-plane system:

6 $$ {G}^{\mathrm{AB}}=\pi r\lambda F\left( r,\lambda \right)\varDelta {G}^{\mathrm{AB}}{e}^{\left(\left({d}_0- d\right)/\lambda \right)} $$

where *d*_0_ is the minimum separation distance between the NM and the surface, taken generally as 0.158 nm for many different kinds of substrates and *d* the separation distance in nanometers.

*G* ^AB^ is defined as a short-range acting potential, having an exponential decrease with the distance. The field of interaction of the potential is mainly determined by the correlation length *λ*, expressed in nanometers. Various values for *λ* have been reported in literature, ranging from 0.2 to 13 nm (van Oss 1993; Wood and Rehmann 2014). The AB interaction can range from distances less than 1 nm up to very few tenths of nanometers and thus compete with the long-range electrostatic and LW potentials. The *F*(*r* , *λ*) term is a function taking into account the shape and the size of the interacting objects. An analytical expression for *F*(*r* , *λ*) between a sphere and a plane can be found in the article from Wood and Rehmann (2014). *F*(*r* , *λ*) depends on the ratio between the radius of the sphere *r* and the correlation length *λ* and tend to the unity when *r* >> *λ*. In this work, we have used relatively large NMs, with *r* > 100 nm, so *F*(*r* , *λ*) can be considered equal to unity.

The nature of the two interacting surfaces intervenes in the AB potential with the term Δ*G* ^AB^ that can be expressed as:

7 $$ \varDelta {G}^{\mathrm{AB}}=-2\ \left(\sqrt{\gamma_N^{\mathrm{AB}}}-\sqrt{\gamma_W^{\mathrm{AB}}}\right)\times \left(\sqrt{\gamma_S^{\mathrm{AB}}}-\sqrt{\gamma_W^{\mathrm{AB}}}\right). $$

where the term $\sqrt{\gamma_i^{AB}}$ refers to the polar component of the surface free energy for the nanoparticle (*N*), water (*W*) and surface (*S*). The values for the $\sqrt{\gamma_i^{\mathrm{AB}}}$ have been calculated using the Owen-Wendt-Fowkes equation (Eq. 8) and determined experimentally with the contact angle between the surface of the collector and two liquids.

8 $$ \sqrt{\gamma_{sv}^{\mathrm{LW}}{\gamma}_{lv}^{\mathrm{LW}}}+\sqrt{\gamma_{sv}^{\mathrm{AB}}{\gamma}_{lv}^{\mathrm{AB}}}=0.5{\gamma}_{lv}\left(1+ \cos {\theta}_y\right) $$

where *s* is solid (surface), *l* is the liquid (water or bromonaphtalene), *v* is the vapour (air) and *θ* is the contact angle.

The potentials were calculated using the function wizard included in the software OriginPro 2015.

## Results and discussion

In the present work, we developed a method for the direct determination of the hydrophobic character of NMs. The hydrophobicity is determined by the direct measurement of the binding affinity of the nanomaterial to the different surfaces or collectors. Each collector is characterised by a combination of surface energy components that according to the XDLVO theory will allow the determination of the surface energy components of the nanomaterials. The affinity is measured by calculating the surface density of nanoparticles immobilised on the specific collector after a given exposure time and after rinsing the sample thoroughly with water to remove loosely bound nanoparticles and possible salt residues.

The detection platform consists in a silicon surface modified with plasma polymer (pAA and PTFE) and layer-by-layer-deposited polyelectrolytes (PSS and PDDA) in order to generate areas with controlled properties. The combination of plasma deposition and polyelectrolytes self-assembly allows the tuning of the surface energy components in a relatively wide range, without dramatically affecting the surface morphology. The selectivity and specificity of the ENMs binding to the surfaces strongly depend on characteristic of the interaction forces such as force strength, range of interaction distance and attractiveness and repulsiveness. Other parameters for the tuning of the interaction forces were ionic strength and pH of the colloidal dispersion used.

### Particles characterisation

Polystyrene particles (200 nm diameter) were used as model hydrophobic NM. The non-modified particles are stabilised by sulfonate groups and are thus negatively charged and hydrophobic. The same types of polystyrene particles modified with carboxyl groups were chosen as hydrophilic model. This surface modification confers to the nanoparticles a higher hydrophilicity.

The hydrodynamic diameter of the nanoparticles was measured by dynamic light scattering together with their zeta potential, which also plays an important role by modifying the electrostatic forces involved in the interactions. To evaluate the difference in hydrophobicity of the different types of NM, their contact angles with water were also measured. All the results are presented in Table 1 for measurements done at pH 7. The PS NMs have a slightly larger hydrodynamic diameter than the PS-COOH while their nominal range declared by the producer is similar (200 nm with a polydispersity index of 10%). The values for the Z-potential indicate that the PS particles, stabilised by sulfonate groups as declared by the producer, are more negatively charged than the PS-COOH particles. The large negative value for the zeta potential enables a colloidal stability for the hydrophobic PS particles even at high salt concentration.

**Table 1:** Characterisation of polystyrene and carboxylate polystyrene particles

	Hydrodynamic diameter (nm) (DLS z-average; pH 7)	Zeta potential (mV)	Contact angle (H_2_O)	Contact angle (α-bromonaphtalene)
PS	236	−67.9 ± 1.5	95 ± 2°	12 ± 5°
PS-COOH	179	−53.7 ± 1.4	23 ± 3°	22 ± 4°

A contact angle of 95° was measured for the PS particle monolayer and 23° for the PS-COOH. Those results confirmed that the modification of the polystyrene particles with carboxyl groups increases the hydrophilicity of the NM, corresponding to a lower contact angle. On the other hand, the contact angle (affinity) for the α-bromonaphtalene was very low for the PS NMs (12°) while increasing (22°) for the PS-COOH particles. Besides, water/octanol partition experiments have been performed and revealed that for both functionalised and non-functionalised materials, no nanoparticles were found in the non-polar octanol phase. This result indicates that this method does not enable to distinguish between hydrophobic and hydrophilic NMs.

### Surface characterisation

To study the selective binding of NMs onto chemically modified surfaces, two sets of surfaces have been prepared. A first set of Si surface has been coated with a plasma-deposited layer of PTFE (hydrophobic) first, and then with several layers of polyelectrolytes (PE) (PSS/PDDA) to enhance the surface hydrophilic character. A second set of surfaces has been prepared with plasma-deposited PAA as starting layer (hydrophilic) and also further modified by the PSS/PDDA multilayers. A complete characterisation was performed on the different substrates to further determine their properties. The results are presented in Table 2.

**Table 2:** Characterisation by zeta potential, ellipsometry, AFM, contact angle and corresponding surface energy components of the modified surfaces: plasma-deposited PTFE (T0), PTFE/PDDA (T1), PTFE/PDDA/PSS (T2), PTFE/PDDA/PSS/PDDA (T3), PTFE/(PDDA/PSS)_2_ (T4), PTFE/(PDDA/PSS)_2_/PDDA (T5) and PTFE/(PDDA/PSS)_3_ (T6) and plasma-deposited PAA (P0), PAA/PDDA (P1), PAA/PDDA/PSS (P2), PAA/PDDA/PSS/PDDA (P3), PAA/(PDDA/PSS)_2_ (P4), PAA/(PDDA/PSS)_2_/PDDA (P5) and PAA(PDDA/PSS)_3_ (P6)

Surface	Zeta potential (mV)	Height (nm)	Roughness (nm)	CA water (deg)	CA bromonaphtalene (deg)	*γ* ^LW^ (mJ/m^2^)	*γ* ^AB^ (mJ/m^2^)
T0	−61.2 ± 0.1	123 ± 1	0.29 ± 0.03	105 ± 1	73 ± 2	19.3 ± 1.9	0.9 ± 0.2
T1	−26.3 ± 0.2	1.24 ± 0.03	0.45 ± 0.05	95 ± 1	65 ± 2	22.3 ± 1.1	2.4 ± 0.1
T2	−60.2 ± 0.9	1.84 ± 0.03	0.48 ± 0.05	84 ± 1	55 ± 2	27.2 ± 1.0	4.9 ± 0.1
T3	−4.9 ± 0.3	2.82 ± 0.02	0.85 ± 0.09	82 ± 2	50 ± 3	29.7 ± 1.5	5.0 ± 0.3
T4	−62.4 ± 0.6	3.41 ± 0.03	0.83 ± 0.08	57 ± 2	43 ± 3	32.9 ± 1.3	16.9 ± 0.5
T5	−4.1 ± 0.3	3.82 ± 0.03	0.85 ± 0.09	72 ± 2	44 ± 4	32.5 ± 1.9	8.6 ± 0.3
T6	−57.6 ± 0.3	4.36 ± 0.03	0.76 ± 0.08	45 ± 2	32 ± 4	37.5 ± 1.5	22.1 ± 0.5
P0	−78.1 ± 1.4	89 ± 1	0.23 ± 0.02	48 ± 1	18 ± 4	41.8 ± 1.0	18.3 ± 0.2
P1	−5.0 ± 0.5	0 ± 0.05	0.12 ± 0.01	43 ± 2	19 ± 4	41.6 ± 1.1	21.2 ± 0.6
P2	−47.4 ± 0.3	1.18 ± 0.18	0.65 ± 0.07	40 ± 2	15 ± 4	42.5 ± 0.9	22.4 ± 0.7
P3	2.8 ± 0.2	2.34 ± 0.17	0.77 ± 0.08	44 ± 3	18 ± 5	41.8 ± 1.3	20.5 ± 1.1
P4	−50.4 ± 0.9	4.41 ± 0.15	1.09 ± 0.11	29 ± 3	12 ± 3	43.0 ± 0.5	27.7 ± 1.2
P5	−6.5 ± 0.7	5.35 ± 0.14	1.88 ± 0.19	33 ± 2	12 ± 4	43.0 ± 0.7	25.8 ± 0.7
P6	−62.6 ± 0.3	7.31 ± 0.17	1.87 ± 0.19	18 ± 4	6 ± 3	43.1 ± 0.3	32.5 ± 2.2

The thickness and the refractive index of the plasma-deposited substrates and the PE layers were measured by ellipsometry after each step of PE deposition. The thickness of the PTFE and the PAA was respectively 123 ± 1 nm and 89 ± 1 nm, with a refractive index of 1.52 ± 0.02 and 1.38 ± 0.04, respectively. The thickness for each PE layer was then fitted using a refractive index of 1.38 (value declared by the producer). The values for the thickness of each PE during the LBL formation are reported in Table 2. The mechanism of formation of the LBL was different for the two polymer substrates: the LBL deposited PE formed on the PTFE is very homogenous (about 0.6 nm/layer) with an initial increase of the roughness (up to 0.85 nm for T3). After the formation of the 4th layer, the value of the roughness remains constant indicating the formation of a homogeneous polyelectrolyte layer. It is most likely due to the exposition of the hydrophobic domains of the first PE layer towards the substrate, leaving the positively charged groups directed towards the water solution. The successive PE mainly interacts through its negative charges to neutralise the positive ones, thus exposing the hydrophobic domains. This mechanism of formation of the PE explains why the T6 sample is not super-hydrophilic (CA water 45 ± 2°) since the external surface contains hydrophobic domains, which contribute to the reduction of the acid-base component of the surface free energy.

The formation of the first positively charged PE layer on the hydrophilic PAA results in an intermixing of positively charged groups and negatively charged carboxyl groups of the PAA. This is demonstrated by a dramatic change in zeta potential (from −78 ± 1 mV to −5 ± 1 mV) and by a slight reduction of the native roughness of the PAA. Furthermore, the thickness of the first layer measured by ellipsometry is lower than 0.3 nm. Subsequently, the surface becomes super-hydrophilic with a relatively large surface energy, and in particular with a relatively large acid-base component.

The AFM analysis shows that the morphology of the collectors is not strongly affected by the formation of the polyelectrolyte layers. The results show that the roughness increases from 0.29 to 0.76 nm for the PTFE substrate and from 0.23 to 1.87 nm for the PAA substrate. This increase in roughness is due to the formation of nano- and micro-clusters of polyelectrolytes during self-assembly. The surface chemical homogeneity at the nanoscale was investigated through AFM with force mapping, using different probes.

**Fig. 1:**
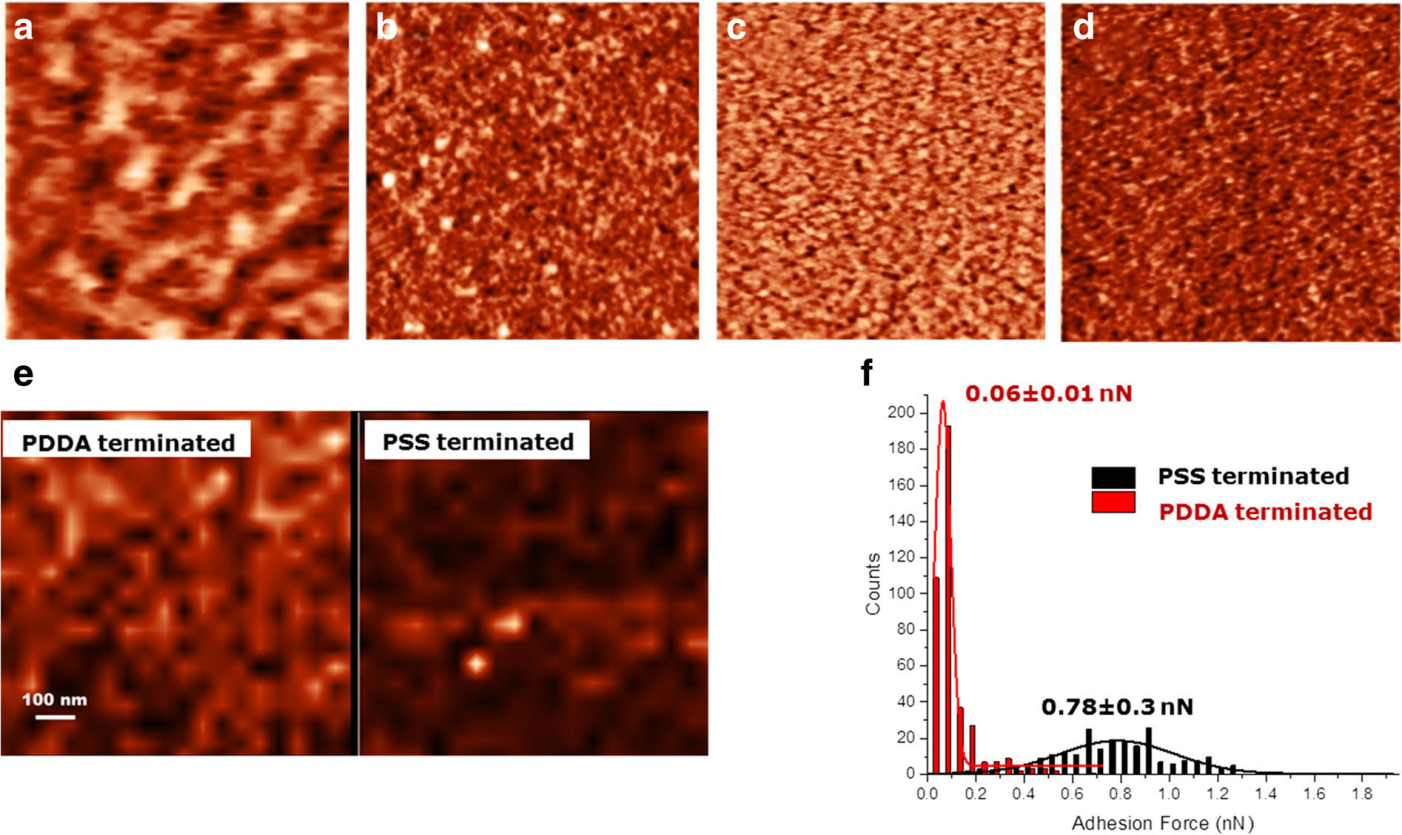
AFM analysis of **a** PTFE, **b** T6, **c** PAA and **d** P6 vertical scales are 0.3 nm for (**a**) and (**c**) and 0.16 nm for (**b**) and (**d**). **e** CFM adhesion maps for a PDDA- and PSS-terminated PTFE substrate measured with a silicon tip, vertical scale is 0.2 nN. The scan size for all the images (**a–e**) is 1000 nm; the colour scale goes from *black* corresponding to the minimum value to *white* corresponding to the maximum value. **f** Statistical distribution of the adhesion forces on the two surfaces

The adhesion force is mapped automatically by the instrument in the selected area with a resolution of 10 nm (the lateral resolution of the technique is limited by the radius of curvature of the used tip, which is nominally 10 nm). The force mapping revealed that the average adhesion of the PDDA-terminated layers was considerably higher than the average adhesion for the PSS-terminated layer, with an average adhesion value of 0.06 ± 0.01 nN for PSS vs. 0.78 ± 0.3 nN for PDDA. The observed increase of roughness was attributed to the formation of clusters due to the intermixing between the two polyelectrolytes during the multilayer formation. The most important information, which could be extracted from these experiments, was that the surfaces were homogeneous at the nanoscale in terms of adhesion forces, and hence they could be treated as flat homogenous plane in the modelling of the interaction forces with the nanoparticles. The results are shown in Fig. 1.

**Fig. 2:**
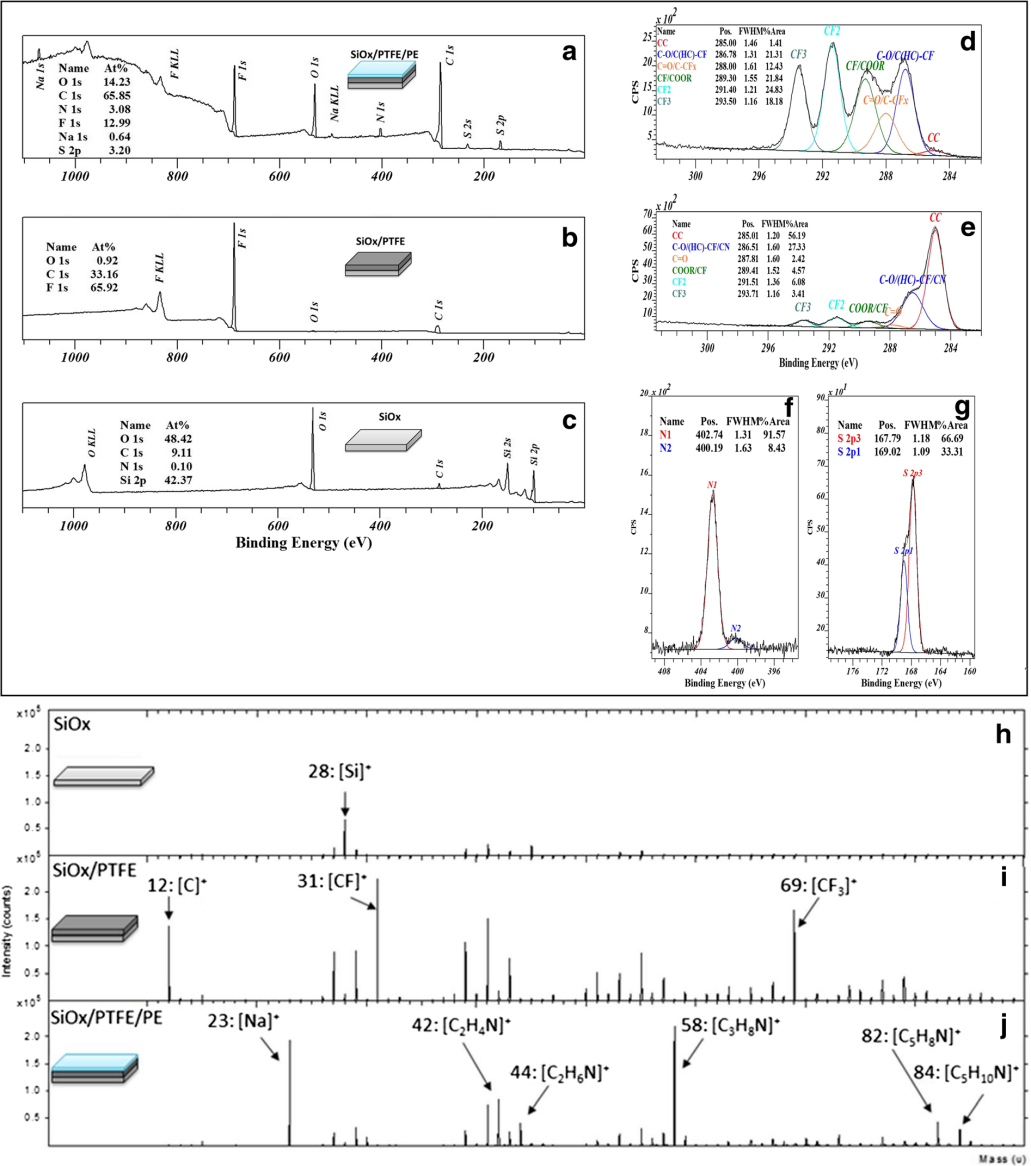
XPS and ToF-SIMS spectra of the substrates used in this work.(*top*): XPS survey spectra of **a** bare SiO_*x*_ and **b** PTFE film and five polyelectrolyte layers deposited on the PTFE film (**c**); C1s core-level spectra of PTFE film (**d**) and PTFE + polyectrolyte layers (**e**); S2p and N1s core level spectra of PTFE + polyectrolyte layers (**f**, **g**). *Bottom*, positive portion of ToF-SIMS spectra of SiO_*x*_ bare substrate (**h**), plasma-polymerised PTFE film (**i**) and polyectrolyte layers deposited on PTFE (**j**)

The chemical composition of the surface was studied by XPS and ToF-SIMS after the surface modifications by the PTFE film and layer-by-layer polyelectrolytes deposition. The spectra are presented in Fig. 2. The surface analysis through XPS measurements after plasma deposition is mainly characterised with a fluorine and a C1s peak having a characteristic PTFE shape (Balazs et al. 2005; Jaszewski et al. 1999) demonstrating the presence of a confluent PTFE layer that masks fully the silicon substrate. The ToF-SIMS static spectrum, which reveals chemical information about the outermost layer thanks to the high surface sensitivity of the technique, confirmed the XPS findings by the identification of carbon and carbon-fluorine clusters such as CF_2_^+^ and CF_3_^+^. The analysis after the six PE layer self-assembly showed the full coverage of the PTFE surface plasma. In fact, from the XPS survey spectra (Fig. 2a–c), it is possible to notice a drastic decrease of the signal given by the PTFE, such as fluorine, and the appearance of nitrogen and sulphur signal, belonging to the PDDA and PSS, respectively. Moreover, a comparison of the C1s core-level spectra collected on the plasma-polymerised PTFE film before and after the deposition of the polyectrolyte layers (Fig. 2d, e) reveals a strong decrease of the components related to the C-F moieties with a corresponding increase of the hydrocarbon, carboxyl, ester and amino compound components. The ToF-SIMS data support the XPS results as can be seen in Fig. 2i, j where positive spectra of the different substrates are illustrated. The appearance of the fragments such as^+^ (31 *m*/*z*) and CF_3_^+^ (69 *m*/*z*) and the corresponding disappearance of the fragments related to the SiO_*x*_ substrate after the PTFE deposition demonstrate that the film is uniform and pin-hole free. After the deposition of the PDDA and PSS, the PTFE fragments and the detection of C_*x*_H_*y*_N_*z*_^+^ mass peaks (e.g. C_3_H_8_N at 58 *m*/*z*) with the correspondent suppression of the peaks related to PTFE further vouching the successful functionalisation of the PTFE film with polyelectrolyte multilayers with a thickness greater than 2 nm. Those analyses demonstrated a good coverage of the substrate, indicating that each polyelectrolyte layer is covering the one underneath, and after siz layers (3 PDDA + 3 PSS alternatively) the PTFE substrate is not detectable.

The surfaces were then characterised in terms of surface energy components using the two-liquid contact-angle technique and using the model of Owen, Wendt and Fowkes, also known as the OWRK theory. Briefly, we measured the advancing contact angle with water and α-bromonaphtalene, respectively. This combination of testing liquids allowed to take into account the polar component (AB) and the dispersive component (LW) of the surfaces. The surface energy components (Lishfitz-van der Waals and acid-base) were then calculated from the contact angles with the two liquids, solving a system of equations in two variables (Eq. 6). The surface energy components for the different collectors are shown in Table 2.

It can be observed that PTFE exhibits the lowest value for both LW and AB components (19.3 mJ/m^2^ and 0.9 mJ/m^2^, respectively). The presence of PE layers results in an increase of the LW and the AB components, with the AB component being larger on the PSS-terminated layers than on the PDDA-terminated layers. On PAA, the surface energy components are 41.8 mJ/m^2^ and 18.3 mJ/m^2^, respectively, and the addition of PSS and PDDA does not change these values dramatically.

To summarise this section, the experiments performed on the PTFE- and PAA-modified surfaces showed the same trend, even though the base layer of PTFE is hydrophobic and that of PAA more hydrophilic. We could indeed reach similar surface properties, with an increase in hydrophilicity on both substrates thanks to the formation of PE layers, a slight increase in roughness and a negative zeta potential for all conditions. The 6th layer enabled to obtain close surface properties with two substrates of different given properties, PTFE or PAA. In conclusion, the LBL-modified hydrophobic and hydrophilic polymers exhibit, after a given number of modification layers:

1. Relatively low surface roughness (RMS roughness <2 nm).
2. Surface chemical homogeneity, as indicated by the XPS and Tof-SIMS analysis and with homogeneous adhesion forces, as indicated by AFM.
3. A negative surface zeta potential (in order to avoid electrostatic attraction forces).
4. Two “wettable” surfaces exhibiting different combination of LW and AB components: T6 and P6 with a relatively low and very high value of the AB component, respectively.

### Nanoparticle-binding study

#### Hydrophobic nanoparticles

**Fig. 3:**
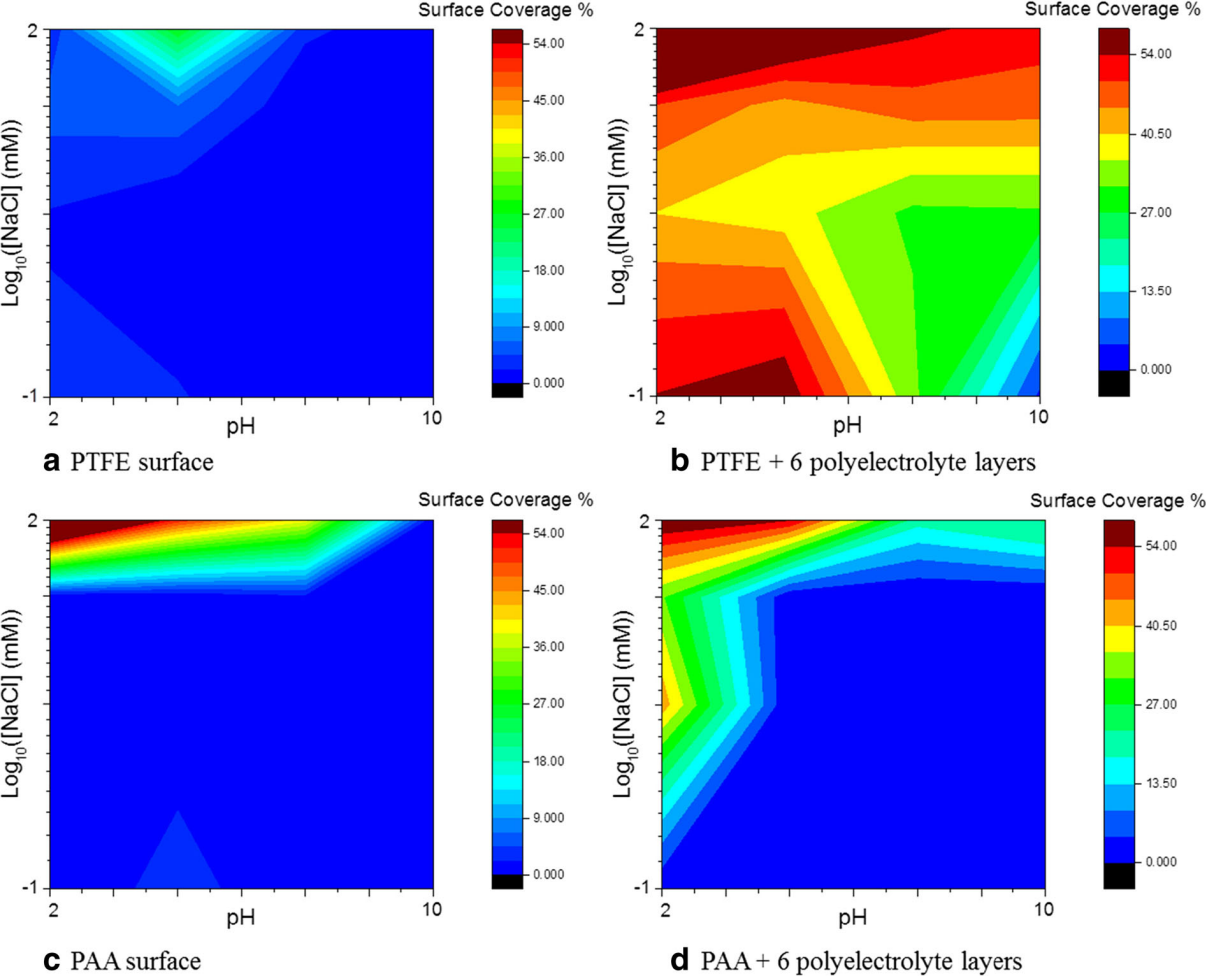
Map of the surface coverage of PS particles on **a** PTFE surface (contact angle, 105°), **b** PTFE modified with six polyelectrolyte layers (contact angle, 45°), **c** PAA (contact angle, 48°) and **d** PAA modified with six polyelectrolyte layers (contact angle, 18°). The colour scale corresponds to the surface coverage in percentage, with *dark blue* being close to 0 and *dark red* to 55%

To evaluate the binding capacity of the hydrophobic collector, a PTFE-coated surface, characterised by a contact angle of 105°, was tested against hydrophobic PS particles. The PS particles were incubated on the surface using 16 different conditions of pH and ionic strength, ranging from 0 to 100 mM NaCl and pH 2 to 10. The goal was to assess the influence of electrostatic forces on NP binding. The surfaces were then analysed via SEM, and the surface coverage was determined for the different conditions using ImageJ software. The results are presented as colour maps in Fig. 3.

The surface coverage of the hydrophobic particles on the hydrophobic collector was found to be low for all conditions, with a slight trend for a higher binding for low pH and high salt concentration. This result could be attributed to the poor wettability of the PTFE surface. Indeed, because of its high hydrophobicity, the immersion of the substrate in water may promote the formation of micro-bubbles (Attard 2003; Steitz et al. 2003), thus creating a physical barrier that impedes the contact between surface and particles in suspension and preventing the hydrophobic interactions to occur. Experiments using surface plasmon resonance was performed in order to confirm this hypothesis; this experiment and the results are presented in the Supporting information.

The modification of the PTFE surface by a multilayer of polyelectrolytes influences the properties of the substrate by drastically lowering the surface hydrophobicity (as shown by the decrease of contact angle down to 45° with water). Such a difference suppresses the formation of a physical barrier when in contact with water (SI.1). The same experiments were then performed with the same set of conditions of ionic strength and pH, with the PTFE surface covered by six layers of PE, which exhibit the lowest contact angle possible for this substrate. The SEM observation of the surfaces following the incubation of the particles showed an important NM binding in all conditions. The mapping of the surface coverage calculated with ImageJ under the different conditions is shown in Fig. 3b.

Compared with the results without PEs, the surface covered by the particles is dramatically increased.

Moreover, one can observe an important increase of the NM binding with the decrease in pH and the increase of salt concentration (red areas on the map 3b) since these experimental conditions favour the screening of the electrostatic repulsive forces. At the opposite, at low ionic strength and high pH, the electrostatic repulsive forces are preponderant impeding the NM to approach the surface leading to a very low binding rate. By screening the repulsive forces, hydrophobic particles can approach and bind on the PTFE covered by six PE layers, demonstrating that the hydrophobic forces generated by the hydrophobic properties of the substrate act at a long range overcrossing the polyelectrolyte layers (Meyer et al. 2006).

Further experiments were performed using the hydrophilic substrate (PAA), with and without PE modification to compare the binding rate obtained with the hydrophobic substrate and to verify the validity of the theory of the long-range interactions in our experimental conditions. The degree of hydrophobicity of the PAA-modified surfaces was tuned from around 48° of contact angle without polyelectrolyte to 18° with six layers of polyelectrolytes. The different surfaces were analysed by SEM after incubation of hydrophobic polystyrene particles in the conditions previously described. The results are presented in the maps in Fig. 3 in terms of surface coverage for PAA alone (Fig. 3c) and PAA with six layers of polyelectrolytes (Fig. 3d). The SEM images are available in Supporting information S.2.

As it can be observed for both PAA alone and PAA + PE surfaces, the surface coverage is extremely low for most conditions, with salt concentration between 0 and 10 mM and for all pH on PAA alone and pH 4 to 10 on PAA + PE. The trend already observed before, with an increase in the binding rate with the increase of ionic strength and decrease of pH is even more evident than before since particle binding only increases in those extreme conditions.

Comparing the results on the PAA and PTFE substrates without polyelectrolytes (Fig. 3a, c), it can be assumed that, with the highest ionic strength, the binding is more important on PAA than on PTFE because of the physical barrier existing due to the highly hydrophobic properties of PTFE. The main binding difference occurs between PTFE + PE and PAA + PE (Fig. 3b, d). Indeed, the addition of PE layers induced a large increase in the binding onto the hydrophobic substrate, whereas the binding change with polyelectrolyte on PAA is observed only for pH 2 with salt. This shows that the hydrophilic superficial layer on hydrophobic substrate allows long-range hydrophobic interactions to take place, resulting in the binding of the hydrophobic particles only onto the hydrophobic substrate.

To summarise, when the hydrophobic PTFE substrate was in direct contact with the aqueous medium containing the nanoparticles, an air interface was generated, limiting the contacts with water, and thus preventing the physical interaction with particles. The most favourable conditions for the binding of hydrophobic particles onto the hydrophobic substrate is obtained by using a superficial hydrophilic layer on top of the hydrophobic substrate and by minimising the electrostatic repulsion thanks to low pH and high salt concentration. The hydrophilic layer has to be thin enough (here around 5 nm) to allow long-range hydrophobic interactions to take place. The use of a polyelectrolyte layer was in this case a good method to obtain a high hydrophilicity with a thin coverage of the hydrophobic substrate. The superficial hydrophilicity degree, representing the wettability of the substrate, was therefore not driving the binding but enabling the hydrophobic interactions, present only with the underlying hydrophobic substrate PTFE to take place.

#### Hydrophilic particles

**Fig. 4:**
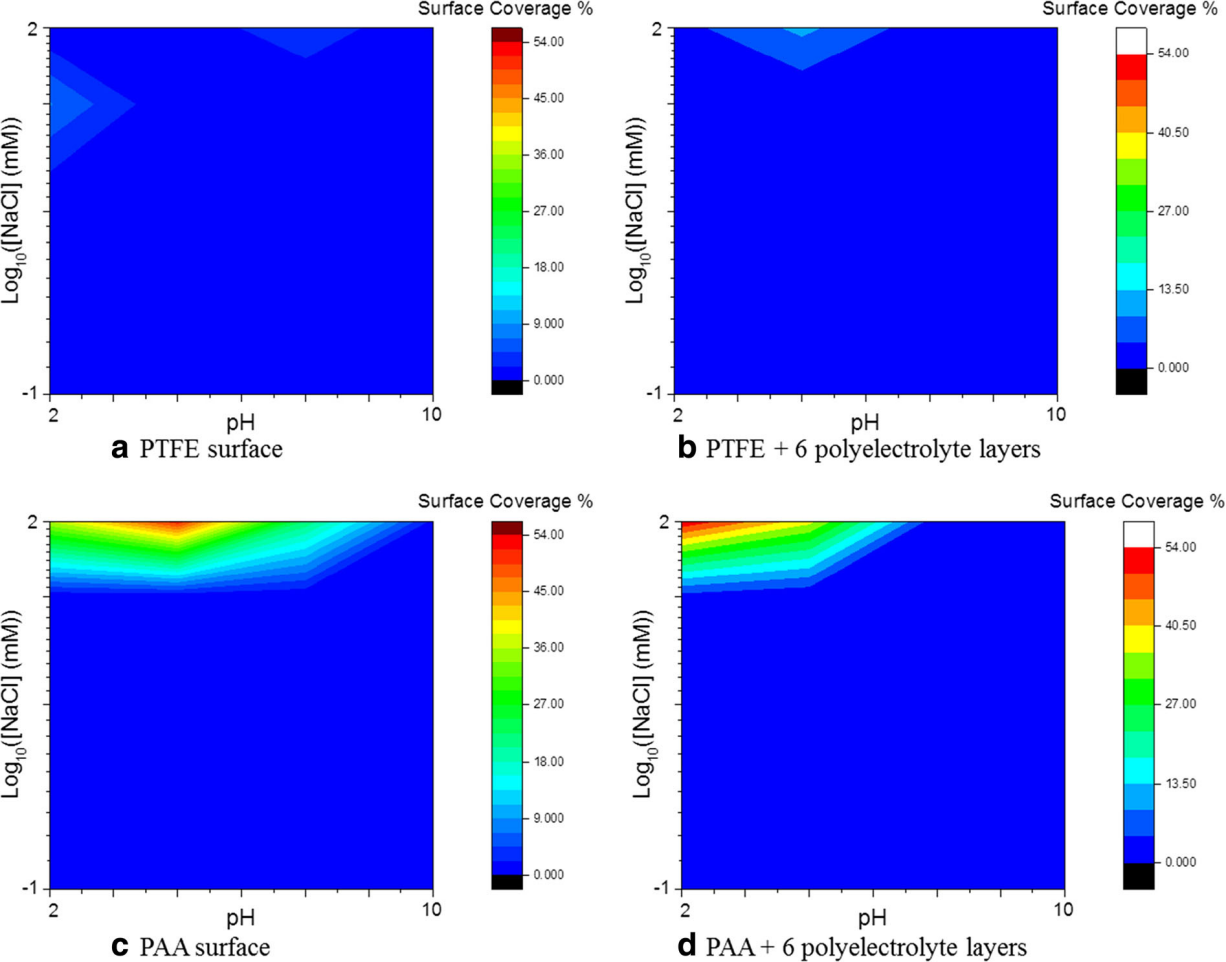
Map of the surface coverage of PS-COOH particles on **a** PTFE surface (contact angle, 105°), **b** PTFE modified with six PE layers (contact angle 45°), **c** PAA (contact angle, 48°) and **d** PAA modified with six PE layers (contact angle, 18°). The *colour scale* corresponds to the surface coverage in percent, with *dark blue* being close to 0 and *dark red* to 55%

To assess the selectivity of the collector towards hydrophobic particles, the same experiments were performed with the particles presenting a higher hydrophilicity. For this purpose, polystyrene particles modified with carboxyl groups were used, providing them hydrophilic properties as shown by a contact angle value in water of 23° and a zeta potential of −53.7 mV. The PS-COOH particles dispersed in 16 conditions of pH and ionic strength were incubated on the different surfaces. The analysis by SEM enabled to calculate the surface coverage for the different conditions. The results are presented as a colour map in Fig. 4.

As it can be seen in Fig. 4, the hydrophilic particles have an extremely low binding rate (<1% of surface coverage in most of the cases) for all conditions on the hydrophobic substrate with or without modification by the polyelectrolyte layers. A significant binding is observed only for high salt concentration and low pH, similar on the modified and non-modified surface. These results confirmed the absence of hydrophobic interactions explaining why no particle is bound to the different surfaces.

From the systematic study of NM binding onto the different collectors, it is possible to determine the surface energy components for the nanoparticles using the XLDVO theory.

### Evaluation of the interaction potential using the XLDVO theory

The experimental observation enabled us to detect the particles exhibiting a good affinity for the surface. We measured the surface density of the particles firmly adsorbed onto the different surfaces after a fixed incubation time. This value is a function of the affinity of the different particles for the different collectors, i.e. for the corresponding interaction potential, which depends on the surrounding solution conditions (pH and salt concentration). The XDLVO equation of the interaction potential between particles and surfaces enables to predict, in the different conditions, if the formation of the particle-surface interface is energetically favourable and if potential barriers are present at different distances from the surface. The potential barriers are able to physically repulse the particles from the surface, influencing their observed affinity for the collectors’ surfaces.

Moreover, only PSS-terminated collectors (negatively charged) were used for the determination of the NM surface properties in order to avoid misinterpretations of the adhesion due to electrostatic absorption between the amines of the PDDA-terminated collectors, with the PS NM having a negative zeta potential. The model presented here consists in the interaction of a hard (not deformable) sphere, with the nominal radius of the NMs, approaching by diffusion a flat and homogenous surface (characterised by the surface energy components measured by the contact angle technique). No interactions between spheres are taken into account.

**Fig. 5:**
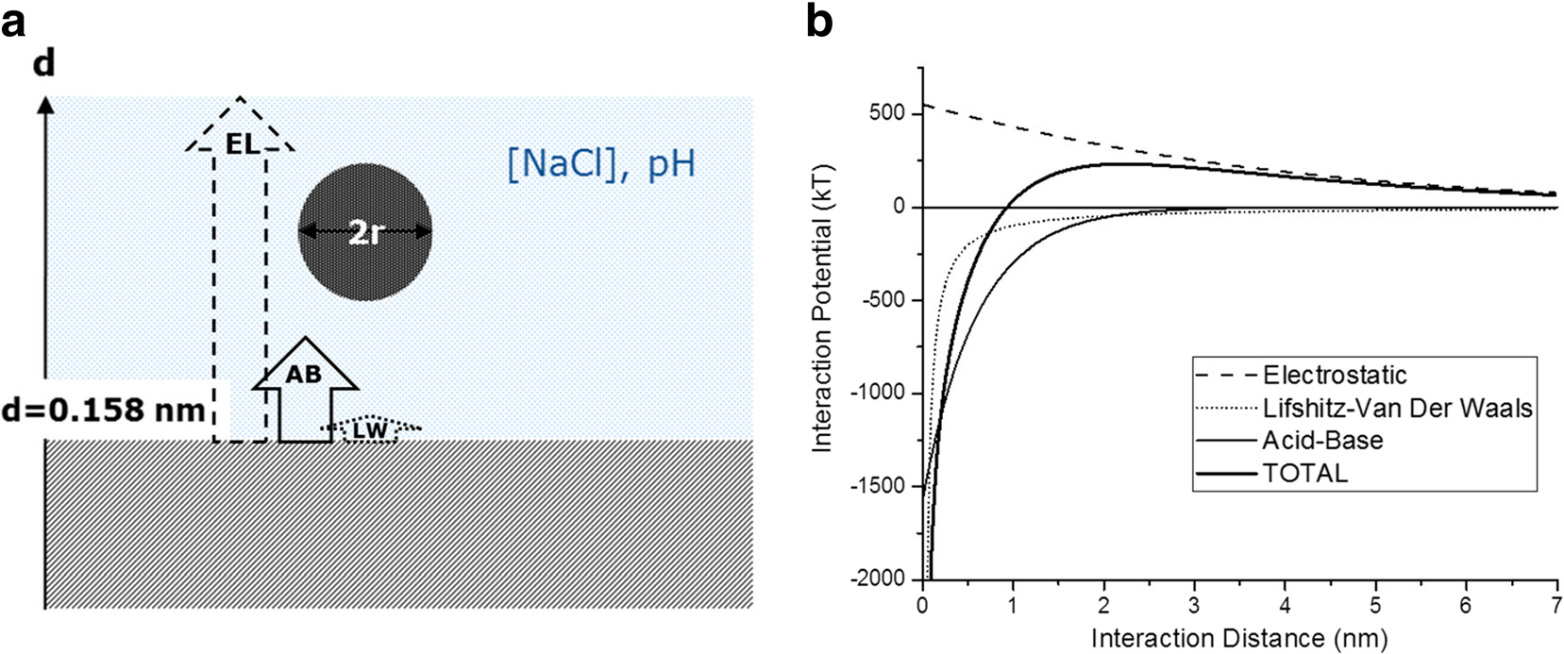
LW, electrostatic and AB potentials for a 200-nm diameter hard sphere, negatively charged (*Z*_pot_ = −52 mV) interacting with a plane (collector) with *Z*_pot_ = −40 mV in NaCl solution 10 mM. Both sphere and collector have *γ*_LW_ = 27 mJ/m^2^ and *γ*_AB_ = 6 mJ/m^2^

The XDLVO potentials as a function of the distance for a sphere-surface physical model have been calculated, and the results are shown in Fig. 5. The contribution of the different forces to the resulting potential is also shown as separated curves. The electrostatic potential between two surfaces of the same charge is always repulsive and corresponds to positive values of energy (kT). The potential barrier is already active at 10 nm and increases close to the surface. On the other hand, the VdW potential is acting at very short distances and is always attractive, with a strength of attraction regulated by the Hamaker constant, which depends on the particle-surface-medium system, and is directly related to the dispersive (LW) components of the interacting entities. With only these two potentials, the hydrophilic/phobic character of the surfaces or in other words their polar contribution to the surface energy is not taken into account. This polar interaction potential is represented by the AB potential, which depends on the polar component (AB) of the surface energy of the particle, of the surface and the medium. The AB potential is exponentially decreasing from the surface with a typical decay length (*λ*) of 0.6 nm, as reported in literature (van Oss 1994).

The AB potential in Fig. 5b is calculated for a hydrophobic spherical surface interacting with a relatively hydrophilic surface in water. Under these conditions, the AB potential is mainly contributing with an attractive force at about 1 nm distance from the surface. This potential is able to decrease the potential barrier created by the electrostatic potential, increasing the affinity of the particles for the surface. The action of the AB potential is able to explain the observed differences in affinity between the different particles with the used collectors. If we compare the interaction potential at pH = 7 and 10 mM [NaCl] (Fig. 5), we observe that for *z* < 1 nm (so very close to the surface), the potential barrier for the hydrophilic NMs (COOH) with both collectors is positive (around 350 kT). The same is occurring for the interaction potential between the hydrophobic particles (PS) and the pAA-6PE surface (highly hydrophilic). The only potential exhibiting a negative value (−500 kT) at that distance range is the one calculated between the PS particles and the PTFE-6PE. This potential is dominated in this range by the AB component as already explained.

**Fig. 6:**
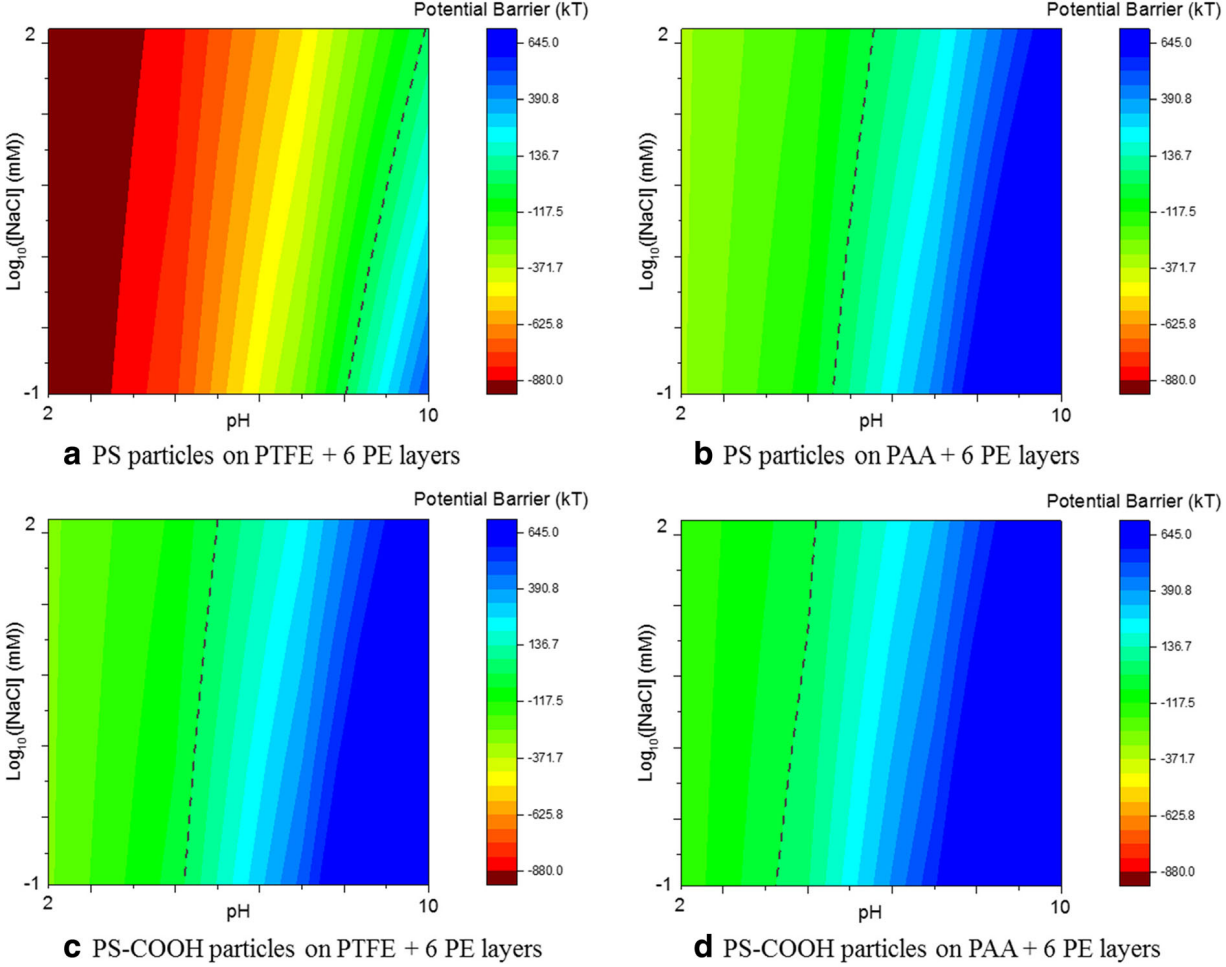
2D colour maps for the value of the potential barrier computed at 0.5 nm between a hard sphere (PS particles in (**a**) and (**b**) and PS-COOH in (**c**) and (**d**)) and a flat surface (PTFE + 6 PE layers in (**a**) and (**c**); PAA + 6 PE layers in (**b**) and (**d**) as a function of the pH and salt concentration using the XDLVO theory. The *green areas* represent the conditions where the energy barrier is negative and the association of the NM with the surface is favourable; whereas the *dashed line* represents the limit where kT = 0

By plotting in a colour map the value of the interaction potential between the particles and the surfaces as a function of the pH and salt concentration, we obtain a direct mapping of the affinity of the particles with the different surfaces (Fig. 6). The areas in red represent the conditions (of salt concentration and pH) at which, at a distance of 0.5 nm from the surface, the potential is negative and consequently the stable adsorption of NMs is favourable. On the other hand, the areas in blue represent a barrier of potential hindering the adsorption of NMs. At pH 7 and [NaCl] = 10 mM, the potential is negative only for the PS (hydrophobic) particles in contact with the PTFE-6PE surface.

The model is therefore in a good agreement with the experimental results, enabling to explain the different affinities of the hydrophobic PS particles compared with the hydrophilic ones for the surfaces characterised by relatively low values of the surface free energy (as the PTFE plus six PE layers). This method can then be used to rapidly and qualitatively characterise the NM hydrophobicity and to empirically evaluate the effect of the surface functionalisation of NMs on their surface properties, which have a tremendous effect on the NM interaction with biological media, the formation of the corona and the subsequent bio-response and toxicity.

## Conclusion

The results obtained in this study demonstrate the proof of concept of a method for sorting NMs according to their hydrophobicity through specifically functionalised surfaces and thanks to the long-range character of the hydrophobic forces involved. To do so, we studied the interactions between substrates with tuned hydrophobicity level with hydrophobic and hydrophilic NMs. The results show that nanoparticles with very different properties (hydrophobic or hydrophilic) have different affinities for these surfaces. In particular, the most hydrophobic NMs show a high affinity for a surface composed of a hydrophobic substrate covered by six layers of PE: a mildly hydrophilic surface (CA 43°), enabling to detect only the hydrophobic NMs in particular ambient conditions (salt concentration and pH). The results are in accordance with the XDLVO model. The determination of the affinity of NMs towards substrate surfaces with different hydrophobicity degrees will enable the direct characterisation of the NMs with unknown surface functionalisation and residual hydrophobicity in a simple and high-throughput way.

Starting from this proof-of-concept study, an in-depth study is envisaged in order to test other types of NM and to improve the detection technique with the aim of achieving a real-time detection. Associated with a real-time detection technique, this method for the detection of hydrophobic NMs will become straightforward and very simple compared with others used in the literature, thanks to the direct and rapid visualisation of the NM’s binding affinity for the collectors.

It can have interesting applications in the field of characterisation of nanomedicine or consumer products containing engineered NMs.

## Electronic supplementary material

ESM 1 (PDF 1621 kb)
