# Supplementary material for: Characterisation of nanomaterial hydrophobicity using engineered surfaces
Source: J Nanopart Res. 2017 Mar 20;19(3):117. doi: 10.1007/s11051-017-3804-z (PMC5357669; doi:10.1007/s11051-017-3804-z)
Supplement: Supplementary file 1 — (PDF 1621 kb) [file 11051_2017_3804_MOESM1_ESM.pdf]

"Characterisation of nanomaterial hydrophobicity using engineered surfaces"

Desmet et al.

Supporting Information

S1. Evaluation of the presence of the air depletion layer on the hydrophobic surface

Surface Plasmon Resonance Imaging (Horiba, SPRi-Plex) has been used to characterise the possible presence of an air layer at the PTFE-water interface that would impedes the nanoparticle adsorption to the surface. PTFE surfaces coated by a 6 layer polyelectrolytes (referenced as T6 in the manuscript) have been used as hydrophilic surface for comparison. The difference in wettability of these two surfaces has been determined by contact angle with water (Table 2 in the main text).

A SPRi standard Gold coated prism was modified with a 20 nm thick PTFE layer deposited using the experimental conditions described in the main text. Then, half of the prism surface was coated by 6 polyelectrolyte layers (T6) and the prism was inserted in the liquid cell of the SPR instrument for studying the interactions of the two surfaces with water.

In Figure S1a the plasmon curves which represent reflectivity as a function of the angle of incidence for the PTFE and the T6 are reported. The results show that the plasmon dip i.e. the minimum of the reflectivity for the T6 surface is shifted towards smaller angle as compared to the plasmon dip of the PTFE surface. This shift toward smaller SPR dip angle is the result of the higher refractive index of water interface in the case of the T6 as compared to PTFE.

In order to determine if the shift could be due to the presence of air bubbles or film at the Water PTFE interface, the same experiments have been performed by using mixture of 85% EtOH – 15% Water and pure EtOH. EtOH has been chosen because its low surface tension i.e. high wettability properties. The corresponding measured plasmon dips are reported in Figure S1b and S1c. Measurements with EtOH result in a different trend. The plasmon curves of T6 and PTFE surfaces are overlapping confirming that the solutions containing EtOH wet perfectly the surface as showed by the contact angles measurement (very small contact angles for both surfaces Table ST1).

Table ST1. Liquid used for the calibration of the refractive index response of the two surfaces.

**Liquid**	**Refractive Index**	**Contact Angle with PTFE °**	**Contact Angle with T6 °**
Water	1.3330	105	45
85% EtOH – 15% Water	1.3569	< 10	< 10
EtOH	1.3611	< 10	< 10

T6 surfaces having a good wettability properties with the three used liquids, the angle of incidence has been plotted versus the refractive index to build a calibration curve (Figure SI1e and the relative linear fitting).

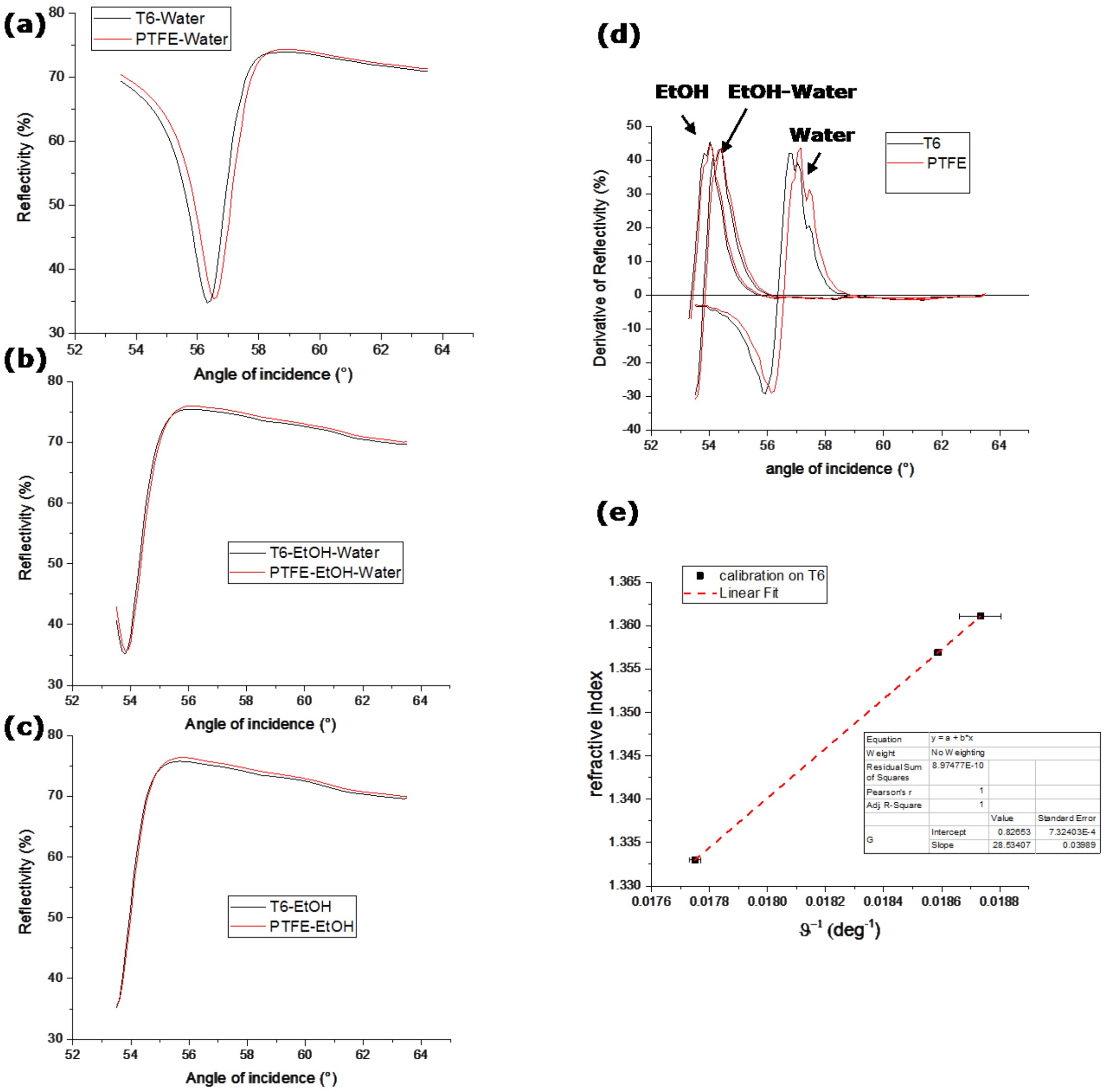

Figure SI1. (a)(b) and (c) plasmon curves for the PTFE and the T6 with respectively water, water-Ethanol mixture and Ethanol (d) first derivative of the curves in (a,b,c) to show the position of the zero which corresponds to the minimum of the plasmon dip (e) calibration curves of the effective refractive index of the upper layer for the two surfaces

The calibration curve can be fitted with the following function:

$n = 0.82653 + 28.53407/\theta$ Eq. SI1

This function can be used to determine the effective refractive index of the layer above the PTFE surface by using the related angle of plasmon dip in Eq. SI1:

$n_{mix} = 1.3309$

This refractive index being lower, it indicates a water layer with a lower density at the interface, which has been interpreted as a layer of sub-micrometer Air bubbles in Water (1). As a first approximation, the proportion of air and water in the mixture can be calculated from the formula:

$n_{mix} = f_{air}*n_{air} + f_{water}*n_{water}$ Eq. S2

where f_{air}, n_{air} and f_{water}, n_{water} represent the filling factor and the refractive index or respectively air and water. From Eq. S1 we obtain:

f_{air} = 0.67 % and consequently f_{water} = 99.33%.

By assuming a constant evanescent wave extending above the gold surface at a distance of 500 nm, we can roughly estimate the thickness of the air layer:

λ_{air} = 500*0.0067 = 3.35 nm

The present calculation assume a continuous air film but the interface can be as well formed a non - homogenous film consisting in bubbles.

The presence of air nano bubbles at the interfaces between a hydrophobic surface and the water has been directly measured by other researchers and with different techniques, reporting a layer of air bubbles with a thickness between 2 and 5 nm[1, 2].

This thin air layer creates a new Air-Water interface immediately above the PTFE surface, which is not present for the T6, due to the presence of the hydrophilic PEL multilayer. The model used for the calculation of Eq. S2 is shown in Figure SI2.

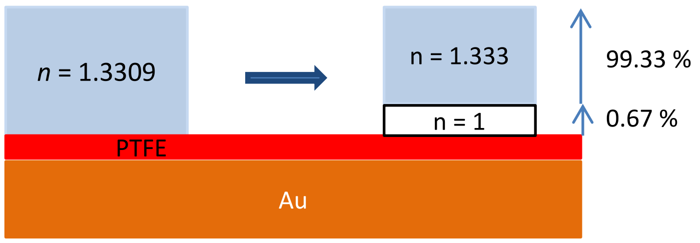

Figure SI2. Model used for the direct measurement of the air layer on top of the PTFE using the SPR-I technique.

The experimental results show that the PS nanoparticles cannot cross the physical barrier of the air layer and remain confined within the water phase. At the opposite, with the PEL coated PTFE surface, the water wets perfectly the surface enabling the particle adsorption as a results of the attractive forces of the surface.

[1]. Steitz, T. Gutberlet, T. Hauss, B. Klösgen, R. Krastev, S. Schemmel, A. C. Simonsen and G. H. Findenegg, *Langmuir*, 2003, 19, 2409-2418.

[2]. D. A. Doshi, E. B. Watkins, J. N. Israelachvili and J. Majewski, *Proceedings of the National Academy of Sciences of the United States of America*, 2005, 102, 9458-9462.

S2. Scanning electron Microscope images of the collectors after PS nanoparticles incubation and rinsing

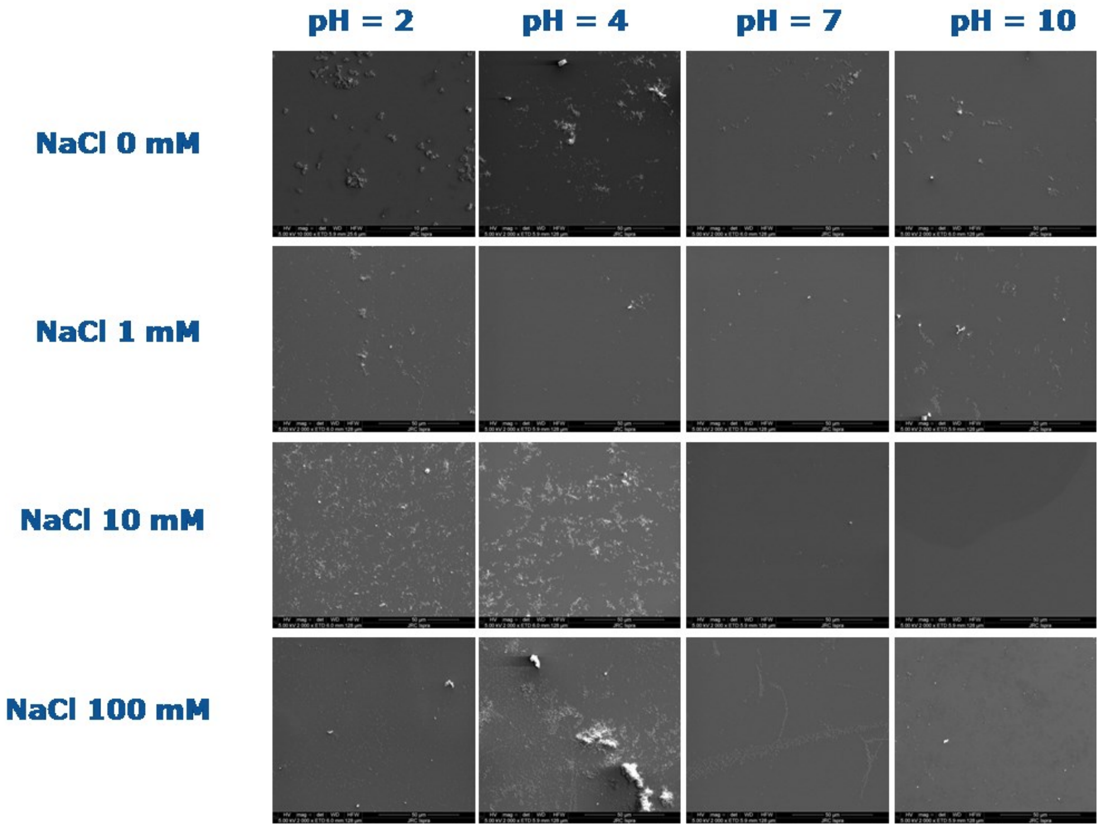

Figure SI3. SEM images of the PS particles on the PTFE collector at different ambient conditions

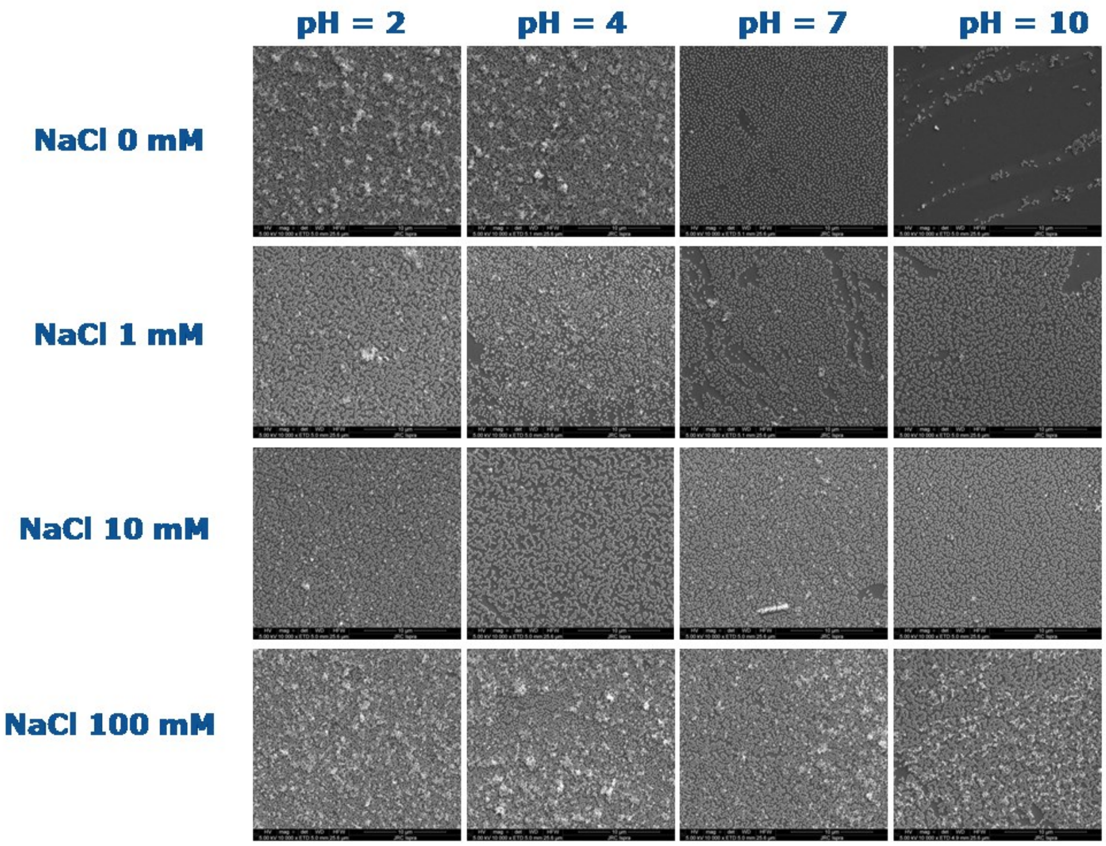

Figure SI4. SEM images of the PS particles on the T6 (PTFE+6PEL) collector at different ambient conditions

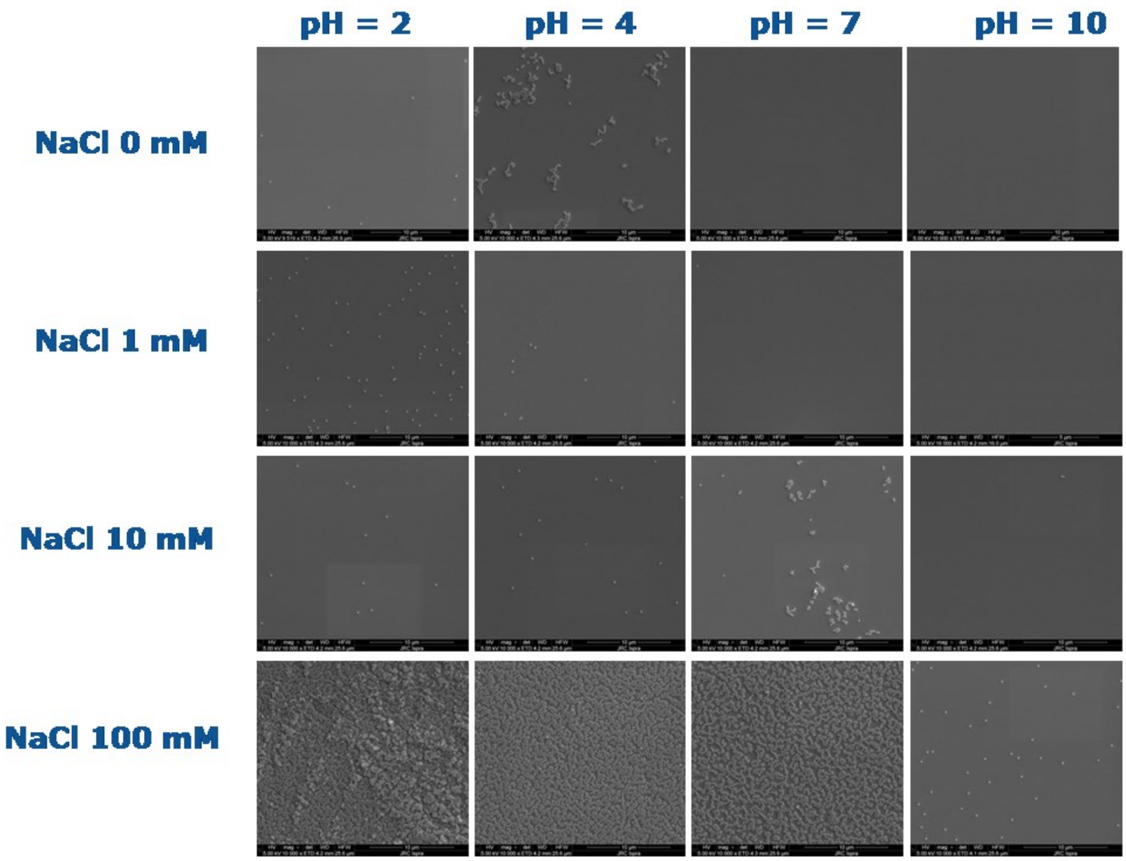

Figure SI5. SEM images of the PS particles on the PAA collector at different ambient conditions

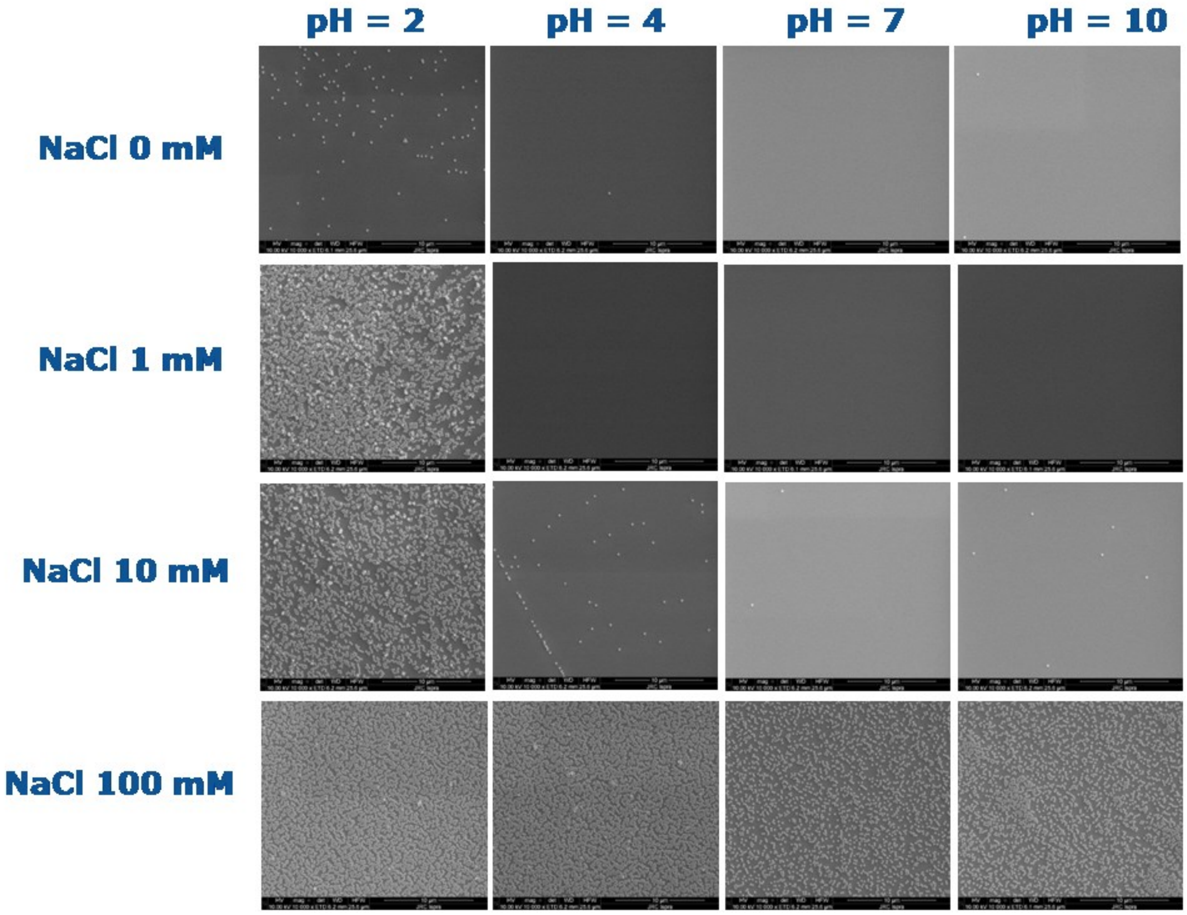

Figure SI6. SEM images of the PS particles on the P6 (PAA+6PEL) collector at different ambient conditions

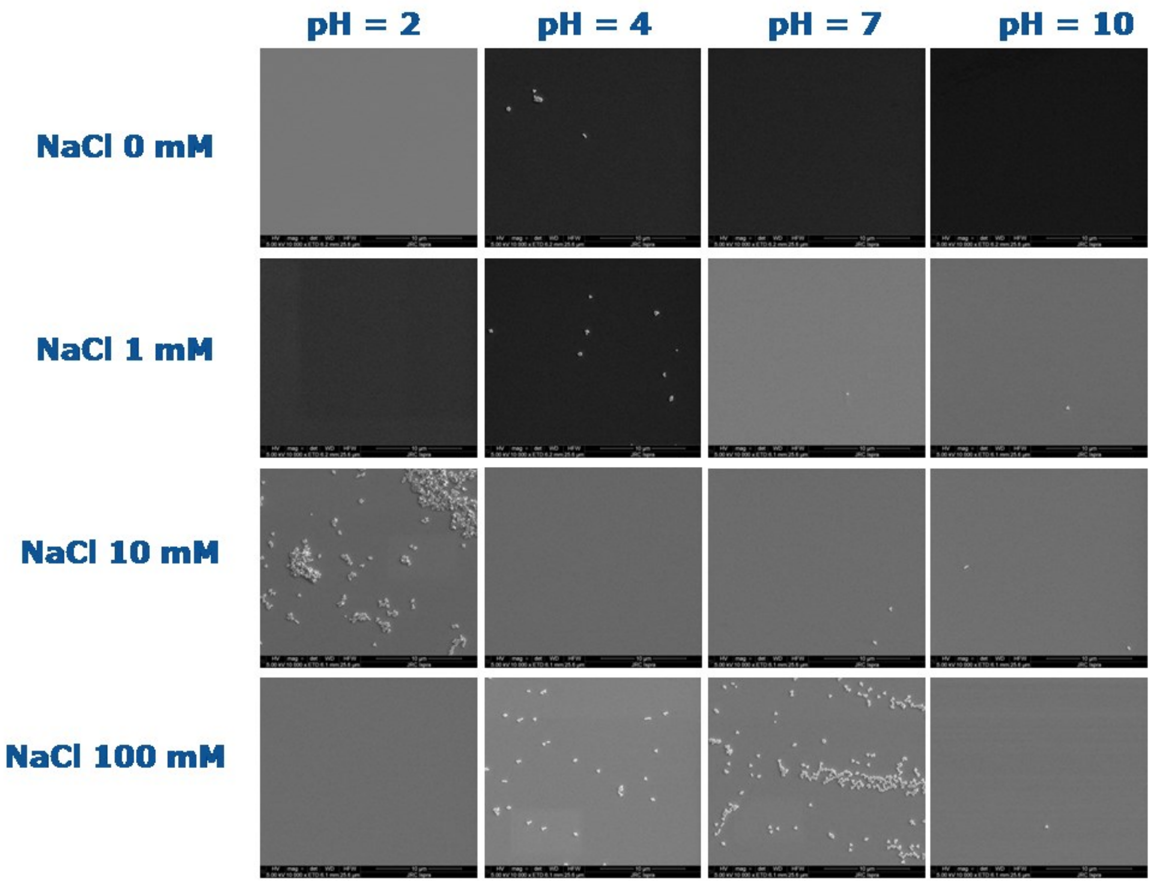

Figure SI7. SEM images of the PS-COOH particles on the PTFE collector at different ambient conditions

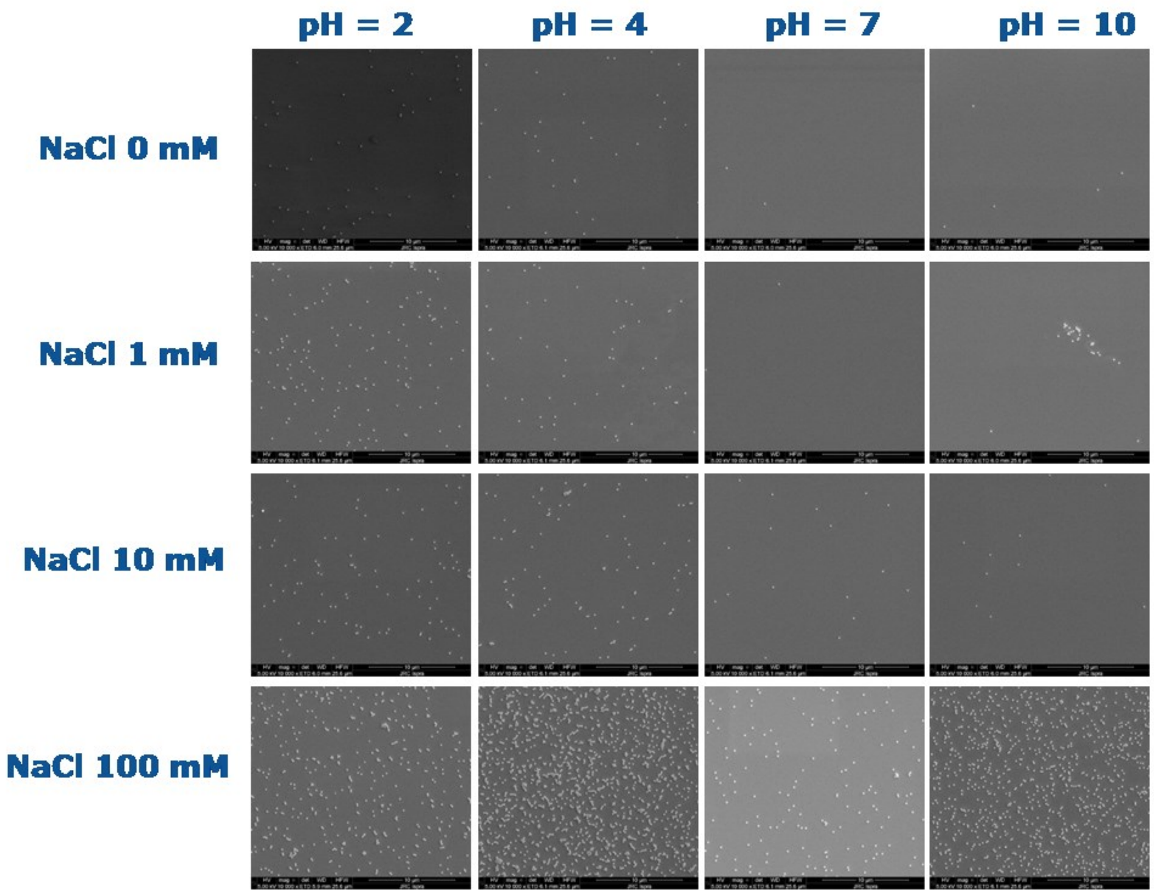

Figure SI8. SEM images of the PS-COOH particles on the T6 (PTFE+6PEL) collector at different ambient conditions

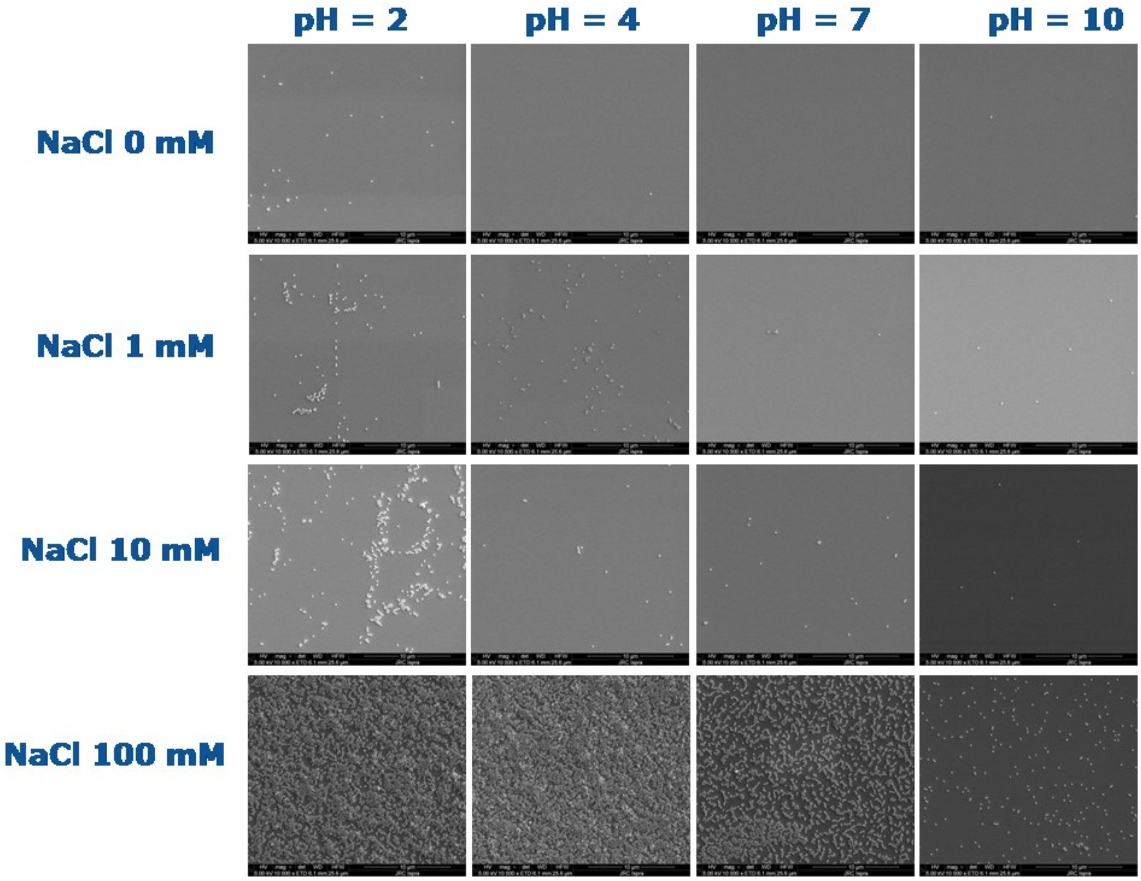

Figure SI9. SEM images of the PS-COOH particles on the PAA collector at different ambient conditions

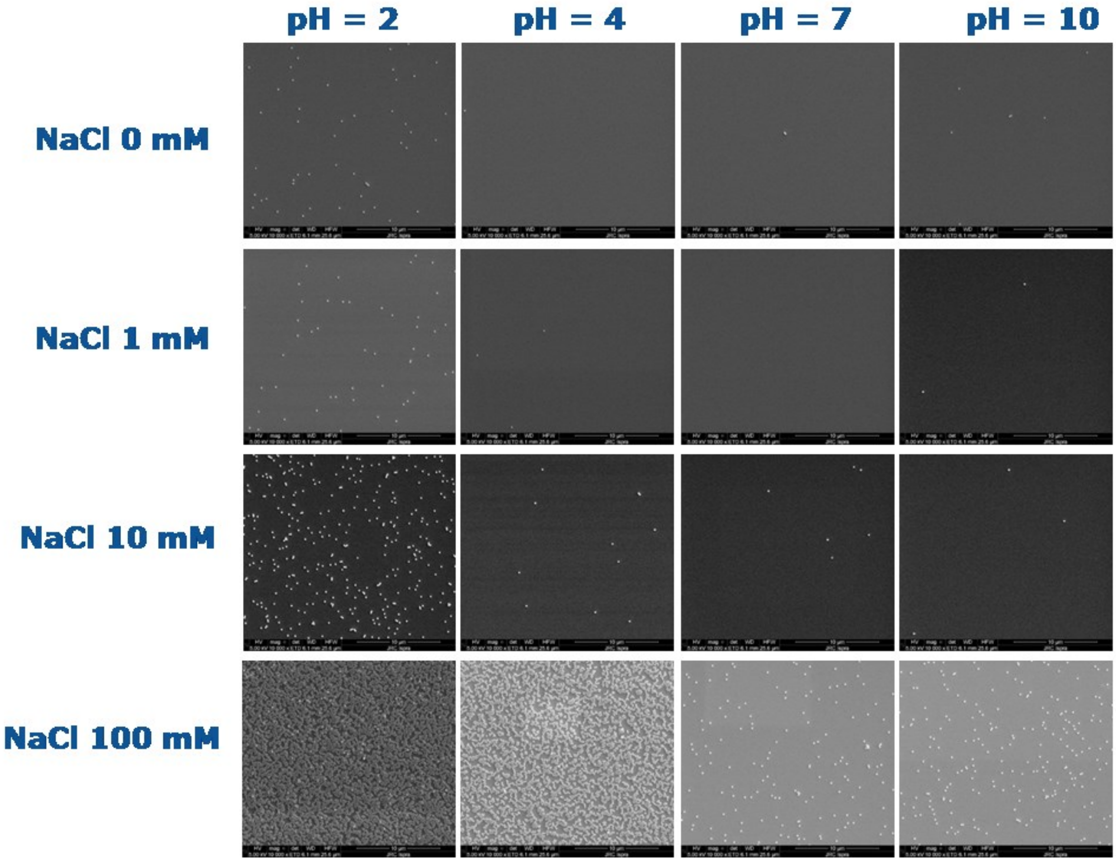

Figure SI10. SEM images of the PS-COOH particles on the P6 (PAA+6PEL) collector at different ambient conditions
